# Rivaling the World's Smallest Reptiles: Discovery of Miniaturized and Microendemic New Species of Leaf Chameleons (*Brookesia*) from Northern Madagascar

**DOI:** 10.1371/journal.pone.0031314

**Published:** 2012-02-14

**Authors:** Frank Glaw, Jörn Köhler, Ted M. Townsend, Miguel Vences

**Affiliations:** 1 Zoologische Staatssammlung München, München, Germany; 2 Hessisches Landesmuseum Darmstadt, Darmstadt, Germany; 3 Department of Biology, San Diego State University, San Diego, California, United States of America; 4 Division of Evolutionary Biology, Zoological Institute, Technical University of Braunschweig, Braunschweig, Germany; University of Lausanne, Switzerland

## Abstract

**Background:**

One clade of Malagasy leaf chameleons, the *Brookesia minima* group, is known to contain species that rank among the smallest amniotes in the world. We report on a previously unrecognized radiation of these miniaturized lizards comprising four new species described herein.

**Methodology/Principal Findings:**

The newly discovered species appear to be restricted to single, mostly karstic, localities in extreme northern Madagascar: *Brookesia confidens* sp. n. from Ankarana, *B. desperata* sp. n. from Forêt d'Ambre, *B. micra* sp. n. from the islet Nosy Hara, and *B. tristis* sp. n. from Montagne des Français. Molecular phylogenetic analyses based on one mitochondrial and two nuclear genes of all nominal species in the *B. minima* group congruently support that the four new species, together with *B. tuberculata* from Montagne d'Ambre in northern Madagascar, form a strongly supported clade. This suggests that these species have diversified in geographical proximity in this small area. All species of the *B. minima* group, including the four newly described ones, are characterized by very deep genetic divergences of 18–32% in the *ND2* gene and >6% in the 16S rRNA gene. Despite superficial similarities among all species of this group, their status as separate evolutionary lineages is also supported by moderate to strong differences in external morphology, and by clear differences in hemipenis structure.

**Conclusion/Significance:**

The newly discovered dwarf chameleon species represent striking cases of miniaturization and microendemism and suggest the possibility of a range size-body size relationship in Malagasy reptiles. The newly described *Brookesia micra* reaches a maximum snout-vent length in males of 16 mm, and its total length in both sexes is less than 30 mm, ranking it among the smallest amniote vertebrates in the world. With a distribution limited to a very small islet, this species may represent an extreme case of island dwarfism.

## Introduction

Extremes in nature such as gigantism and dwarfism in organisms attract considerable attention from the general public, but also allow biologists to gain general insights into morphological and ecological constraints. While the largest animals are generally well known, miniaturized species often go undetected, and striking new discoveries of dwarf species are not uncommon. Among endotherm amniotes, minimum body size is typically limited by the mass/surface-area relationship, because maintenance of a constant body temperature requires an increasing proportion of the energy budget as this ratio decreases. Even the smallest bats, shrews and hummingbirds reach body lengths of at least 30–50 mm. Among ectothermic amniotes, the gecko lizard species *Sphaerodactylus ariasae* is the smallest yet described, with a snout-vent length (SVL) of at most 18 mm and a total length (TL) of 33 mm [1]. The smallest species of snake is the Lesser Antillean threadsnake (*Tetracheilostoma carlae*; formerly in the genus *Leptotyphlops*) with a total length of about 100 mm [2].

Miniaturization has been postulated to constitute an important pre-adaptation for evolutionary novelties that may lead to the evolution of entirely new patterns of organismal organization [3]. Miniaturized tetrapods are often characterized by reduced and simplified adult morphologies; often the diminutive species resemble juveniles or subadults of larger related taxa [3]. Miniaturization is also sometimes associated with an evolutionary loss of skull bones and phalangeal elements, and with other features such as relatively larger braincases. Such features probably often reflect functional constraints and paedomorphosis [4]. As a practical point, the reduction in externally visible characters in miniaturized species can also pose problems for alpha taxonomy [5].

*Brookesia, Calumma*, and *Furcifer* constitute the three genera of chameleons occurring on Madagascar, an island which harbors almost 80 of the world's ca. 185 nominal chameleon species [6]–[8]). All three genera are endemic to Madagascar, with the exception of two *Furcifer* species on the nearby archipelago of the Comoros [9], [10]. *Calumma* and *Furcifer* are generally medium-sized to large arboreal chameleons (ca. 50–295 mm SVL and 110–695 mm TL), and are often vividly coloured [7]. On the contrary, the 26 currently recognized species of *Brookesia* typically dwell and forage on the ground, often within the leaf litter on the floor of rainforest and dry deciduous forest, and climb at night to low perches in the vegetation for sleeping. They are characterized by a typically dull brown or (rarely) greenish colour, a short non-prehensile tail that is used as “fifth leg” in walking [11], and a smaller size of ca. 15–65 mm SVL and 25–105 mm TL [7].

This ecomorphological disparity among chameleons is also found in mainland Africa, leading to use of the general terms “tree chameleons” and “ground chameleons [10].” African ground chameleons comprise the genera *Rhampholeon* and *Rieppeleon*, and are superficially quite similar to the Malagasy *Brookesia* (which are often further distinguished from other ground chameleons by the name “leaf chameleons”). Interestingly, neither ground chameleons as a whole, nor the African species, form monophyletic groups. In fact, *Rieppeleon* forms the sister taxon of a tree chameleon genus (*Archaius*) endemic to the Seychelles islands [10], indicating a history of multiple independent evolutionary shifts between arboreal and terrestrial chameleon ecomorphs.

Most species of *Brookesia* have very small distribution ranges [6], [12]; indeed, almost 50% of the species are known from single localities [13]. Within *Brookesia*, both species diversity and levels of endemism are highest in northern Madagascar, and are correlated with elevational and environmental heterogeneity in this area [12], [14]. *Brookesia* appears to form the sister clade of all other chameleons [10],[14],[15],[16] which has led to speculation of a Malagasy origin of chameleons [17]. Molecular and morphological phylogenetic data indicate that *Brookesia* can be divided into three major clades [14]: *B. lolontany* and *B. nasus*, two moderately-sized species (SVL = 28–49 mm, TL = 43–87 mm) with laterally compressed bodies and long snouts, form the most divergent clade. A second clade consists of about 18 species with more robust bodies and blunted snouts (SVL = 34–55 mm, TL = 56–110 mm). The third clade is the *Brookesia minima* group (as defined by [18]), and contains all of the truly miniaturized *Brookesia*. It originally was composed of five very small species with SVL 14–30 mm and TL 22–48 mm [19]: *Brookesia dentata*, *B. minima*, *B. peyrierasi*, *B. ramanantsoai* and *B. tuberculata*. These species were all characterized by a small to minute body size, and an absence of dorsal ridges or keels, pelvic shields, and cloacal tubercles.

The reduction of external morphological complexity in these miniaturized representatives of *Brookesia* has led to considerable taxonomic confusion. Citing an absence of clear external morphological differentiation, Raxworthy and Nussbaum [12] synonymized *B. peyrierasi* and *B. tuberculata* with *B. minima*, and *B. ramanantsoai* with *B. dentata*. Subsequently, another species of this group was described (*B. exarmata*) [20], and it was demonstrated that *B. peyrierasi* and *B. tuberculata* are valid species, based on differences in both external and genital morphology [5], [20]. Molecular evidence has supported monophyly of the *B. minima* group, albeit with the inclusion of one slightly larger species, *B. karchei* [14]. Further, compared to species within other *Brookesia* clades, all described *B. minima* group species are characterized by unusually high genetic differentiation strongly supporting their specific distinctness.

During recent fieldwork in northern Madagascar, we discovered several new populations assignable to the *B. minima* group, some of which are morphologically and genetically distinct from all described species. We here integrate comprehensive datasets spanning molecular genetics, external morphology, morphometrics, and genital morphology on all *B. minima* group species, with the main goal of taxonomically revising the *B. minima* group and to formally describe four of the newly discovered populations as new species.

## Materials and Methods

Several study sites in northern Madagascar including rainforests and deciduous dry forests on karstic limestone were surveyed for reptiles in the period between 2000 and 2008. Most specimens were collected at night during the rainy season, using torches and headlamps to detect roosting chameleons in the vegetation. Localities were georeferenced with GPS receivers using the WGS84 datum system.

All field research and collecting of specimens were approved by the Malagasy Ministère de l'Environnement, des Eaux et des Forêts (Direction des Eaux et Forêts, DEF) under the following permits: 156-MEF/SG/DGEF/DADF/SCB dated 12 December 2002; 238-MINENVEF/SG/DGEF/DPB/SCBLF dated 14 November 2003; 238-MINENV.EF/SG/DGEF/DPB/SCBLF/RECH dated 22 December 2004; 272-MINENV.EF/SG/DGEF/DPB/SCBLF/RECH dated 8 November 2005; 298-MINENV.EF/SG/DGEF/DPB/SCBLF/RECH dated 22 December 2006; and 036/08 MEEFT/SG/DGEF/DSAP/SSE dated 30 January 2008). Export of specimens was approved by the DEF under permits: 063C-EA02/MG03, dated 26 February 2003; 094C-EA03/MG04, dated 1 March 2004; 103C-EA03/MG05, dated 15 March 2005; E 1400/06, dated 1 June 2006. Import of specimens into Germany was approved by the German authorities (Bundesamt für Naturschutz) under permits: E 1263/05, dated 20 April 2005; E 1561/07, dated 2 May 2007; and No E1481/10 dated 2010.

Voucher specimens were euthanized using approved methods (anaesthesia with ketamine, followed by ketamine overdosis) that do not require approval by an ethics committee. All specimens were fixed in 90% ethanol. Muscle tissue samples for molecular analysis were taken from all specimens and preserved in pure ethanol. Definition of measurements and the description scheme of the holotypes follows previous *Brookesia* descriptions [12]. Several additional characters were also scored, and their definition follows a previous morphological revision of the *B. minima* group [5]. Morphometric measurements were taken by MV with a digital caliper to the nearest 0.1 mm, following a previously published measuring scheme [5]. Measurements taken include: TL; SVL; (these two were previously defined) TAL, tail length; HW, maximum head width; HH, maximum head height; ED, eye diameter; FORL, forelimb length.

Museum acronyms are: Museum National d'Histoire Naturelle, Paris (MNHN), Université d'Antananarivo, Département de Biologie Animale (UADBA); Zoologisches Forschungsmuseum Alexander Koenig, Bonn (ZFMK); Zoologische Staatssammlung München (ZSM). FGZC and ZCMV refer to field numbers of F. Glaw and M. Vences, respectively; MgF refers to Madagascar Frontiers field numbers.

For univariate and multivariate analyses in Statistica version 7 (Statsoft) we combined the new measurements obtained herein with those previously reported [5]. We performed Principal Component Analyses (PCA) based on original morphometric values, separately for males and females, leaving factors unrotated. We calculated selected morphometric ratios as a means for morphometric species distinction, and used non-parametric rank correlation to explore how these ratios are related. Furthermore, separately for males and females of species with sufficient sample sizes, we performed an ANCOVA analyses for all untransformed variables (SVL used as covariable) with subsequent Scheffé Post-Hoc tests for pairwise comparisons among species.

Taxon sampling for phylogenetic analyses included representatives of each divergent lineage (i.e., putative species) known from the *Brookesia minima* clade [14]. Outgroup taxa included *B. superciliaris* and *B. brygooi* (both from the strongly supported sister group to the *B. minima* clade [14]), and also the more distantly related *B. nasus* [14]. Genomic DNA was extracted and amplified using standard protocols [21]. An approximately 570 base-pair (bp) fragment of the mitochondrial protein-coding *ND2* gene was amplified and sequenced for all specimens using the primers 5′ TGA CAA AAA ATT GCN CC 3′ (L4882) [22] and 5′ AAA ATR TCT GRG TTG CAT TCA G 3′ (H5617a) [23]. We furthermore report phylogenetic results based on reduced alignments (but with all major lineages still represented) of the nuclear protein-coding genes *RAG1* (1522 bp) and *CMOS* (846 bp), and the mitochondrial gene for 16S rRNA (*16S*; 580 bp). Sequences of these genes were retrieved from GenBank and complemented for some of the newly described species using primers and methods as used previously [14]. Sequence data for this same fragment has been collected for a wide range of Malagasy reptiles and amphibians, and pairwise divergences of 3–5% or higher have been found to typically characterize pairs of well-defined species (e.g., [24], [25]). Finally, we performed a combined phylogenetic analysis including representatives of all major lineages for all genes. All newly determined DNA sequences were submitted to Genbank (accession numbers JN673976–JN674062). See Supporting Information S1 for museum/collection and GenBank numbers of specimens. We used the Clustal algorithm [26] implemented in the program DAMBE [27] to align all sequences by their amino-acid translations, and adjusted alignments by eye using MacClade 4.03 [28]. Ambiguously aligned regions were excluded from all analyses.

All phylogenetic analyses were performed in a Bayesian framework using the program MrBayes v3.1.2 [29]. Almost all the nuclear sequences were previously used in a larger phylogenetic study of the genus *Brookesia*, where they were analyzed as part of a concatenated multi-gene dataset [14]. The sole exception to this involves a previously unreported *CMOS* sequence from an individual belonging to an undescribed population in the Analamazava forest of northeastern Madagascar, relatively close to the type locality of *B. karchei* in Marojejy. Each gene was partitioned by codon position, and best-fitting evolutionary models for each partition were chosen using the Akaike Information Criterion (AIC) as implemented in MrModelTest [30]. Two separate MrBayes runs (each consisting of two independent analyses) were conducted for each dataset. Within each run, the average standard deviation of split frequencies (ASDSF) and the potential scale reduction factor (PSRF) statistics from MrBayes were used to evaluate topological and branch-length convergence, respectively. Although convergence appeared complete in all cases by 10 million generations, all analyses were run for 20 million generations, and the first five million generations of each were discarded as burn-in on the basis of evaluation of the ASDSF and PSRF statistics. All MrBayes analyses were performed via the CIPRES 2 portal [31] at the San Diego Supercomputer Center.

Bayesian divergence-dating analyses were conducted using BEAST v1.6.2 [32]. Eight extra-chamaeleonid nodes within Lepidosauria were constrained largely as in previous analyses [10] (see Supporting Information S1 for details and references for time constraints). Two separate BEAST analyses were each run for 60,000,000 generations, with sampling every 2000 generations. The first 10,000 trees from each of these runs were discarded as burn-in and they were combined to make the summary tree in Tree Annotator (part of BEAST package). ESS values >200 were achieved for all parameters.

### Nomenclatural Acts

The electronic version of this document does not represent a published work according to the International Code of Zoological Nomenclature (ICZN), and hence the nomenclatural acts contained in the electronic version are not available under that Code from the electronic edition. Therefore, a separate edition of this document was produced by a method that assures numerous identical and durable copies, and those copies were simultaneously obtainable (from the publication date noted on the first page of this article) for the purpose of providing a public and permanent scientific record, in accordance with Article 8.1 of the Code. The separate print-only edition is available on request from PLoS by sending a request to PLoS ONE, Public Library of Science, 1160 Battery Street, Suite 100, San Francisco, CA 94111, USA along with a check for $10 (to cover printing and postage) payable to “Public Library of Science”.

In addition, this published work and the nomenclatural acts it contains have been registered in ZooBank, the proposed online registration system for the ICZN. The ZooBank LSIDs (Life Science Identifiers) can be resolved and the associated information viewed through any standard web browser by appending the LSID to the prefix “http://zoobank.org/”. The LSID for this publication is: urn:lsid:zoobank.org:pub:05FFAF32-FED5-44B2-8844-E58DFD01A233.

## Results

### New locality records of diminutive leaf chameleons

**Figure 1:**
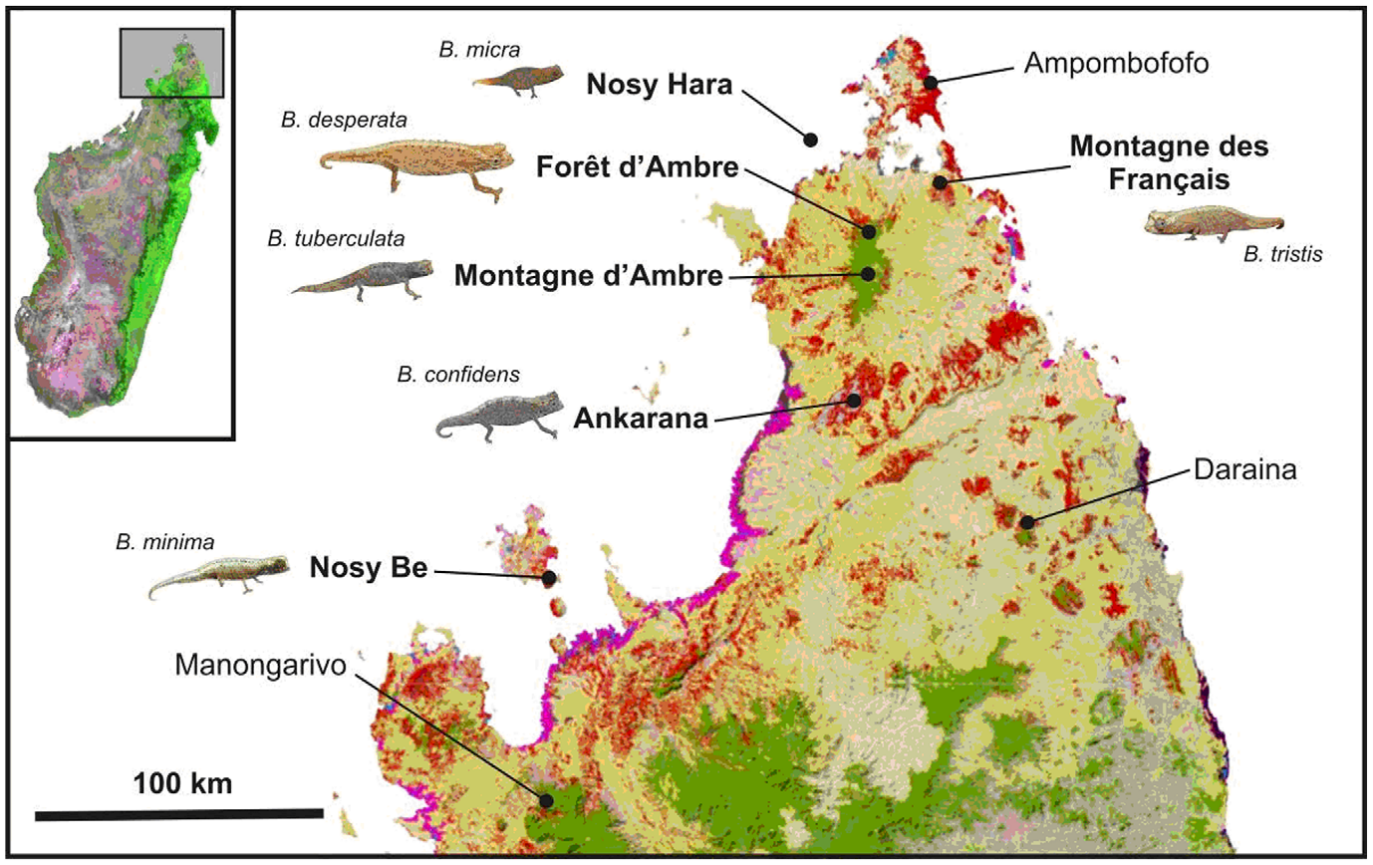
Map of northern Madagascar showing distribution of species of the *Brookesia minima* group. Type localities in bold, *B. dentata*, *B. exarmata*, *B. karchei*, *B. peyrierasi*, and *B. ramanantsoai* not included, because their ranges are located further south; see inset maps in Fig. 2. Orange (dry forest) and green (rainforest) show remaining primary vegetation in 2003–2006, modified from the Madagascar Vegetation Mapping Project (http://www.vegmad.org).

During intensive fieldwork in northern Madagascar, we discovered numerous new populations of leaf chameleons of the *Brookesia minima* group (Fig. 1) relative to the most recent revision [5]). In order to facilitate the taxonomic interpretation of the phylogenetic and morphological analyses in the following sections, we here briefly summarize the new data. Some of these came from other authors, and specimens were not available to us for examination: *Brookesia dentata* was rediscovered in Ankarafantsika, close to its type locality [33], [34] and a molecular analysis [14] of a tissue sample provided by these authors proved its distinctness from *B. ramanantsoai*. Samples from Betampona in the northern central east of Madagascar were likewise genetically highly divergent from all described species [14], but the voucher specimens were juveniles and thus their morphology could not be adequately evaluated. Several samples of *B. karchei* from Daraina provided by A. Yoder clustered with sequences of that species from the type locality Marojejy but had a strong genetic divergence; however, the specimens were not available for morphological examination. The identity of specimens historically assigned to *B. karchei* is in need of revision as well. Additionally, one juvenile specimen from Ampombofofo in far northern Madagascar could not be assigned to any species or candidate species and had no associated tissue sample for molecular analysis, and will here be only briefly discussed on the basis of its morphology.

The following new records were supported by new voucher specimens available to us, and by tissue samples used in molecular analyses (see Fig. 1 for localities): (a) specimens from Manongarivo that morphologically correspond to *B. minima* from its type locality Nosy Be; (b) additional specimens of *B. tuberculata* from its type locality Montagne d'Ambre; (c) additional specimens of *B. peyrierasi* from its type locality Nosy Mangabe; (d) a new, hitherto unknown population of small leaf chameleons from dry forest in the karstic limestone massif Montagne des Français; (e) a new population from dry forests in the karst of Ankarana reserve; (f) a new population of extremely small leaf chameleons from the tiny island of Nosy Hara; and (g) a new population of relatively large leaf chameleons of the *B. minima* group, superficially similar to *B. karchei*, from Forêt d'Ambre, a lowland forest bordering Montagne d'Ambre.

The wealth of new material and the obvious morphological distinctness of some of the new specimens (especially the Nosy Hara population) prompted us to study their genetic differentiation, external morphology and genital morphology in more detail. Our integrative data clearly and concordantly indicate that populations d-g listed above represent new, undescribed species, and anticipating their formal description below, we will in the following sections use the new species names erected herein: *Brookesia tristis* (specimens from Montagne des Français), *B. confidens* (Ankarana), *B. micra* (Nosy Hara), and *B. desperata* (Forêt d'Ambre).

### Molecular phylogeny and molecular differentiation

**Figure 2:**
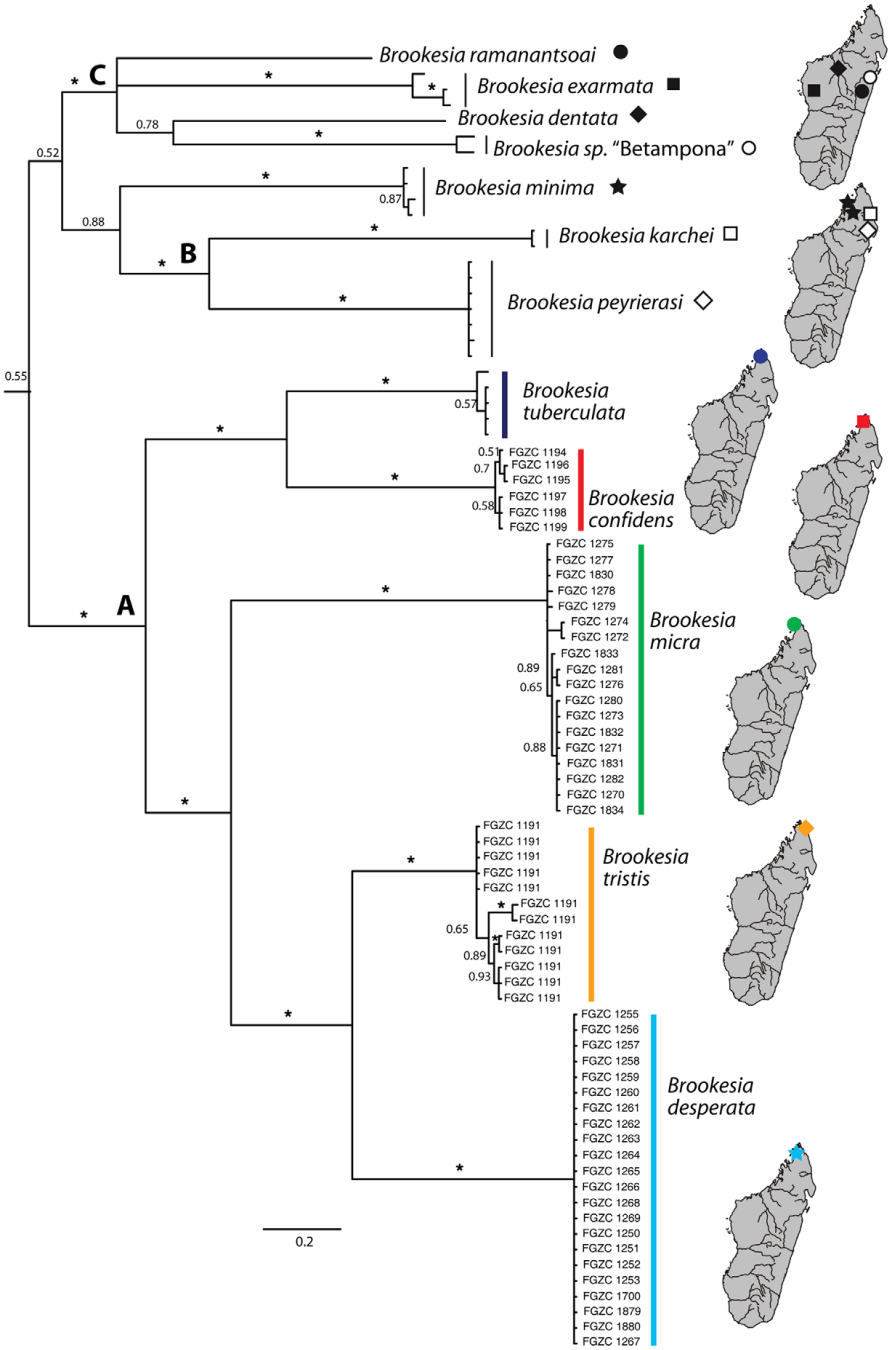
Phylogenetic relationships among species of the *Brookesia minima* group based on mitochondrial DNA sequences. Phylogram (50% majority rule consensus tree) from a Bayesian inference analysis of the 568 bp DNA sequence alignment of the mitochondrial *ND2* gene showing deep divergences among and low differences within species of the *B. minima* group. Species of the northern clade (*B. tuberculata* plus the four new species described herein) are marked in colour. The tree was rooted with *Brookesia nasus*, and including *B. brygooi* and *B. superciliaris* as hierarchical outgroups (not shown). A, B and C refer to major clades as discussed in the text. Inset maps show known distribution ranges (typically single localities) of all species. Asterisks denote posterior probabilities of 1.0.

Sequences from the northern populations described herein form strongly supported clades that are deeply divergent from each other and from the remaining *Brookesia* species (Fig. 2). Support is strong for interrelationships of these clades. *Brookesia confidens* is the sister taxon of *B. tuberculata*. Both taxa form the sister taxon to a clade containing *B. micra*, *B. tristis*, and *B. desperata*, and within this clade *B. tristis* and *B. desperata* are sister taxa.

Average uncorrected *ND2* sequence divergences amongst all lineages identified by this analysis are quite high. *Brookesia confidens* is 20% divergent from its sister taxon *B. tuberculata*, *B. tristis* is approximately 18% divergent from *B. desperata*, and *B. micra* is about 23% and 26% divergent from *B. tristis* and *B. desperata*, respectively. The highest uncorrected pairwise divergences reaches 32.6% between *B. karchei* and *B. micra*, and the smallest interspecific distance is 18% between *B. desperata* and *B. tristis*. The largest intraspecific distances are within *B. exarmata* (3%), *B. tristis* (up to 2.6%), and *Brookesia* sp. from Betampona (2.1%); all other species have maximum divergences among haplotypes ≤1.2%.

Divergences amongst species in the *B. minima* group are known to be uniformly deep (Eocene or Early Oligocene) [14]. Although the *confidens-tuberculata* and *tristis-desperata* divergences are slightly lower than other sister-taxon divergences within the *B. minima* group, they are still larger than almost all sister-taxon divergences in other parts of the *Brookesia* tree [14].

**Figure 3:**
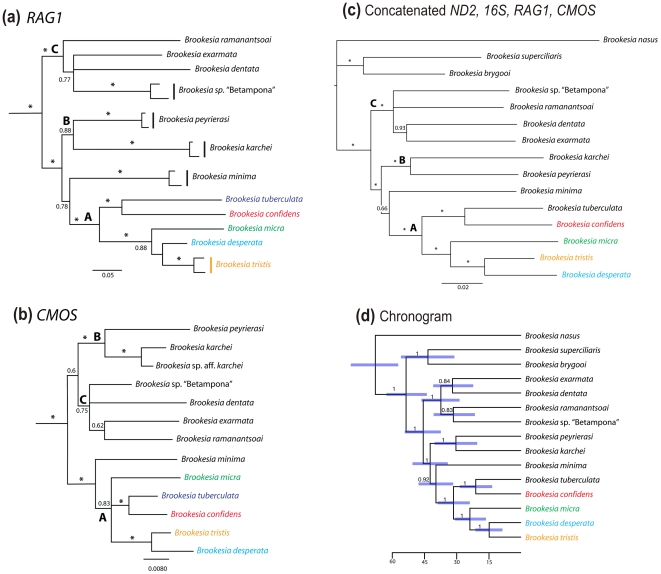
Phylogenetic relationships among species of the Phylograms (50% majority rule consensus trees) from Bayesian inference analyses of DNA sequence alignments of (a) the nuclear genes *CMOS* (846 bp) and (b) *RAG1* (1522 bp). (c) Phylogram from BI analysis of concatenated DNA sequences of the nuclear genes *CMOS* (846 bp) and *RAG1* (1522 bp), and the mitochondrial genes ND2 (568 bp) and 16S (580 bp). (d) *Brookesia* portion of the Bayesian chronogram derived from the BEAST analyses of all four concatenated genes (see Supporting Information S1 for full chronogram with all outgroups). Posterior probabilities are indicated above branches, and bars represent 95% HPDs for mean date estimates. Units on scale are millions of years. Species of the northern clade (*B. tuberculata* plus the four new species described herein) are marked in colour. The trees were rooted with *Brookesia nasus*, and including *B. brygooi* and *B. superciliaris* as hierarchical outgroups (not shown). A, B and C refer to major clades as discussed in the text. Asterisks denote posterior probabilities of 1.0.

With respect to the newly described species, the *RAG1* analysis recovered exactly the same topology as the *ND2* analysis, with almost uniformly strong support (Fig. 3a). Likewise, the *CMOS* analysis recovered the *confidens-tuberculata* and *tristis-desperata* clades with strong support, and showed no strongly supported conflicts with the *RAG1* or *ND2* results (Figs. 2 and 3b). The *confidens-tuberculata* and *tristis-desperata CMOS* divergences were 1.8% and 1.6%, respectively, which once again are at least as high as sister-taxon divergences in other parts of the *Brookesia* tree [14]. In the combined *ND2/16S/CMOS/RAG1* tree (Fig. 3c), relationships among the new species are completely concordant with those from the *RAG1* and *ND2* analyses, and all relevant posterior probabilities are 1.0. Bayesian age estimates confirm previous assessments of very old ages of all species in the *B. minima* group [14]. All sister species split over 10 million years ago, and most of them have ages around 30 mya (Fig. 3d; Supporting Information S1).

Sequence data for the mitochondrial 16S rRNA gene has been collected for a wide range of Malagasy reptiles and amphibians, and uncorrected pairwise divergences of 3–5% or higher have been found to typically characterize pairs of well-defined species (e.g., [24], [25], [35]). A comparison of established species-level 16S divergences and corresponding divergences between lineages discussed here provides additional support for the species status of these newly described populations. The new species described herein show divergences >6% to all other species in the *B. minima* group, with the lowest divergences corresponding to the comparisons *confidens-tuberculata* and *tristis-desperata* (6.7% and 6.4%, respectively).

### Morphological and morphometric differences

**Figure 4:**
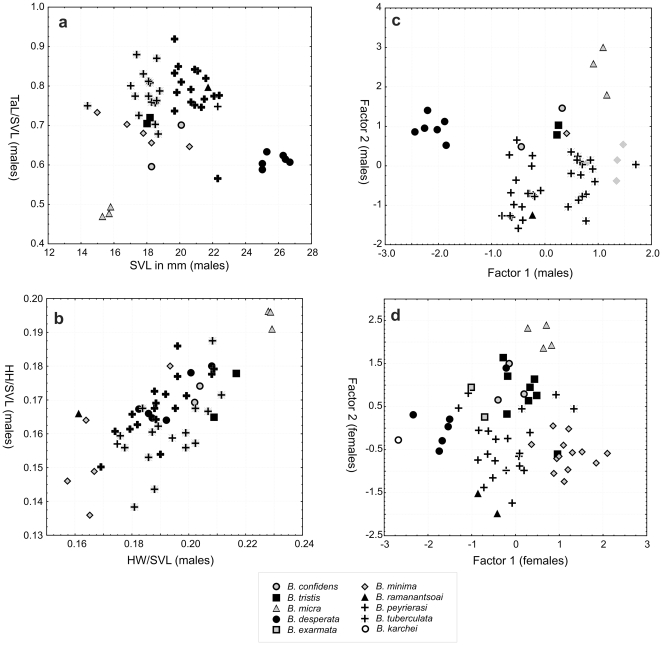
Morphometric differentiation among species of the *Brookesia minima* group. The two left graphs are scatterplots of the first two factors of Principal Component Analyses of (a) male and (b) female specimens of the *Brookesia minima* group based on measurements in Tables 1 and 2, Supporting Information S1 and [5]. Note that Factor 1 is mostly influenced by the size of specimens (see Table 5). The graphs on the right (c–d) are univariate scatterplots of selected measurements and morphometric ratios of male specimens of species in the *Brookesia minima* group.

Despite their superficial similarity, a detailed comparison yielded numerous differences in morphology among species in the *B. minima* group, including those newly identified and delimited herein (Tables 1, 2, 3, 4). Regarding morphometric differences, we here focus on those differences that are diagnostic. Principal component analyses (PCA) of our measurements, analysed separately for males and females (Fig. 4a–b), reveal that by far the majority of the variance (75–80%) is explained by the first factor. This factor had high loadings for all variables, suggesting that it is strongly influenced by body size (Table 5). Factor 2, which is mostly influenced by tail length, and to a lesser degree by eye diameter, explains 11–15% of the variance. Factor 3 explains only 4–5% of the variance, and is mostly influenced by head width, head height and eye diameter, although none of these variables had a particularly high factor loading.

**Table 1:** Morphometric measurements (in mm) of examined type specimens of *Brookesia tristis* and *B. confidens*.

Species/voucher number	type status	Sex	TL	SVL	TaL	HW	HH	ED	FORL
*B. tristis*									
ZSM 1704/2004	HT	M	31.3	18.2	13.1	3.8	3.0	2.1	6.5
ZSM 1705/2004	PT	F	31.4	20.0	11.4	4.0	3.3	2.0	7.0
ZSM 354/2004	PT	F	32.0	18.0	14.0	3.6	3.1	1.8	5.6
ZSM 2146/2007	PT	F	34.8	23.0	11.8	4.4	3.9	2.2	7.2
ZSM 2147/2007	PT	F	35.5	23.8	11.7	4.2	3.7	2.5	7.3
ZSM 2148/2007	PT	F	36.5	23.0	13.5	4.4	3.7	2.1	7.4
ZSM 2149/2007	PT	F	32.2	21.0	11.2	3.9	3.2	2.2	6.7
ZSM 1505/2008	PT	M	30.7	18.0	12.7	3.9	3.2	2.1	5.8
ZSM 1706/2004	PT	F	32.5	20.8	11.7	4.1	3.5	2.1	6.6
ZSM 1707/2004	PT	F	32.9	21.1	11.8	4.0	3.6	2.0	7.0
*B. confidens*									
ZSM 2150/2007	HT	M	29.2	18.3	10.9	3.7	3.1	2.1	6.5
ZSM 2151/2007	PT	F	32.5	20.6	11.9	4.3	3.7	2.4	7.7
ZSM 2152/2007	PT	F	33.3	21.5	11.8	4.2	3.7	2.4	8.1
ZSM 2153/2007	PT	F	36.2	22.6	13.6	4.4	3.8	2.3	7.9
ZSM 1511/2008	PT	F	33.2	21.6	11.6	4.1	3.8	2.0	6.9
ZSM 1512/2008	PT	M	34.2	20.1	14.1	4.1	3.5	2.1	7.4

Used abbreviations: HT, holotype; PT, paratype; M = male, F = female, J = juvenile; TL = total length; TaL = tail length; HW = head width; HH = head height; ED = horizontal diameter of eye; FORL = arm length.

**Table 2:** Morphometric measurements (in mm) of examined type specimens of *Brookesia micra* and *B. desperata*.

Species/voucher number	type status	Sex	TL	SVL	TaL	HW	HH	ED	FORL
*B. micra*									
ZSM 2181/2007	HT	M	23.6	15.8	7.8	3.6	3.1	2.1	5.3
ZSM 2180/2007	PT	J	14.9	10.7	4.2	2.5	2.5	1.3	3.6
ZSM 2182/2007	PT	J	15.0	10.2	4.8	2.5	2.2	1.5	3.6
ZSM 2183/2007	PT	M	23.2	15.7	7.5	3.6	3.0	1.7	5.5
ZSM 2184/2007	PT	F	27.0	18.7	8.3	3.8	3.5	2.0	6.4
ZSM 2185/2007	PT	M	22.5	15.3	7.2	3.5	3.0	2.2	4.8
ZSM 2186/2007	PT	F	27.6	19.3	8.3	3.8	3.6	2.0	7.1
ZSM 2187/2007	PT	F	28.8	19.9	8.9	4.3	3.8	2.2	6.9
ZSM 1509/2008	PT	F	26.9	19.7	7.2	4.0	3.6	2.0	6.6
ZSM 1510/2008	PT	J	15.0	10.6	4.4	2.5	2.3	1.4	4.1
*B. desperata*									
ZSM 2170/2007	HT	M	39.7	25.0	14.7	4.8	4.1	2.5	9.4
ZSM 2171/2007	PT	M	42.7	26.3	16.4	4.8	4.4	2.6	8.1
ZSM 2172/2007	PT	F	46.7	30.0	16.7	5.2	4.6	2.2	9.7
ZSM 2173/2007	PT	F	47.6	30.0	17.6	5.5	5.0	2.7	10.0
ZSM 2174/2007	PT	F	43.3	28.2	15.1	4.9	4.7	2.3	9.4
ZSM 2175/2007	PT	M	41.3	25.3	16.0	4.7	4.2	2.4	9.0
ZSM 2176/2007	PT	M	40.1	25.0	15.1	5.2	4.5	2.6	9.0
ZSM 2177/2007	PT	M	42.6	26.4	16.2	5.3	4.7	2.5	9.1
ZSM 2178/2007	PT	F	43.5	27.3	16.2	5.0	4.5	2.4	9.5
ZSM 2179/2007	PT	M	42.9	26.7	16.2	5.0	4.4	2.6	8.9
ZSM 1506/2008	PT	F	44.9	28.5	16.4	5.0	4.6	2.3	9.9

Used abbreviations as in Table 1.

**Table 3:** Summary of morphometric differences between species in the *B. minima* group.

	*B. tristis*	*B. confidens*	*B. micra*	*B. desperata*	*B. exarmata*	*B. minima*	*B. ramanantsoai*	*B. dentata*	*B. karchei*	*B. peyrierasi*	*B. tuberculata*
N (males)	2	2	3	6	0	5	1	0	0	18	16
N (females)	8	4	4	5	2	13	2	0	1	18	3
TL (males)	31.0±0.4 (30.7–31.3)	31.7±3.5 (29.2–34.2)	23.1±0.56 (22.5–23.6)	41.6±1.4 (39.7–42.9)	–––	29.7±2.9 (26.0–33.9)	39.0	–––	–––	37.3±1.6 (34.2–39.8)	31.8±2.1 (25.2–34.8)
TL (females)	33.5±1.9 (31.4–36.5)	33.8±1.6 (32.5–36.2)	27.6±0.9 (26.9–28.8)	45.2±1.9 (43.3–47.6)	40.0±0.2 (39.8–40.1)	31.2±2.7 (26.0–35.6)	43.2±0.4 (42.9–43.5)	–––	51.0	38.7±3.0 (32.2–43.1)	31.1±3.0 (28.9–34.6)
SVL (males)	18.1±0.1 (18.0–18.2)	19.2±1.3 (18.3–20.1)	15.6±0.3 (15.3–15.8)	25.8±0.8 (25.0–26.7)	–––	17.7±2.1 (15.0–20.6)	21.7	–––	–––	20.9±1.0 (19.7–22.4)	17.9±1.1 (14.4–18.8)
SVL (females)	21.3±1.9 (18.0–23.8)	21.6±0.8 (20.6–22.6)	19.4±0.5 (18.7–19.9)	28.8±0.5 (27.3–30.0)	26.1±0.6 (25.7–26.5)	19.0±1.8 (15.5–21.8)	24.9±0.0 (24.9–24.9)	–––	30.7	23.4±2.2 (19.1–27.4)	19.7±1.8 (18.2–21.7)
TAL/SVL (males)	0.71 (0.71–0.72)	0.65 (0.60–0.70)	0.48 (0.47–0.49)	0.61 (0.59–0.63)	–––	0.68 (0.65–0.73)	0.8	–––	–––	0.78 (0.57–0.92)	0.77 (0.68–0.88)
TAL/SVL (females)	0.56 (0.49–0.77)	0.56 (0.54–0.60)	0.44 (0.37–0.45)	0.58 (0.54–0.59)	0.53 (0.50–0.56)	0.63 (0.59–0.75)	0.74 (0.72–0.75)	–––	0.66	0.66 (0.53–0.73)	0.59 (0.57–0.60)
HW/SVL (males)	0.21 (0.21–0.22)	0.20 (0.20–0.20)	0.23 (0.23–0.23)	0.19 (0.18–0.21)	–––	0.17 (0.16–0.19)	0.16	–––	–––	0.19 (0.17–0.21)	0.19 (0.18–0.21)
HW/SVL (females)	0.19 (0.18–0.20)	0.20 (0.19–0.21)	0.20 (0.20–0.22)	0.18 (0.17–0.18)	0.17 (0.17–0.17)	0.17 (0.15–0.18)	0.16 (0.15–0.16)	–––	0.17	0.19 (0.17–0.20)	0.21 (0.20–0.21)
HH/SVL (males)	0.17 (0.17–0.18)	0.17 (0.17–0.17)	0.20 (0.19–0.20)	0.17 (0.16–0.18)	–––	0.15 (0.14–0.18)	0.17	–––	–––	0.17 (0.15–0.19)	0.16 (0.14–0.19)
HH/SVL (females)	0.16 (0.15–0.17)	0.17 (0.17–0.18)	0.19 (0.18–0.19)	0.17 (0.15–0.17)	0.16 (0.16–0.16)	0.16 (0.14–0.16)	0.17 (0.15–0.18)	–––	0.16	0.17 (0.16–0.19)	0.18 (0.17–0.20)

The table includes only rudimentary data presented for *B. dentata* which is morphologically similar to *B. ramanantsoai* and of which no specimens were examined morphologically by ourselves. Abbreviations as in Table 1. For TL and SVL, data are in mm and are given as mean ± standard deviation, with minimum–maximum in parentheses. Ratios are given as median, with minimum–maximum in parentheses. N is total number specimens examined for morphometry (original data in Tables 1–2, Supporting Information S1 and [5]).

**Table 4:** Summary of morphological differences between species in the *B. minima* group.

	*B. tristis*	*B. confidens*	*B. micra*	*B. desperata*	*B. exarmata*	*B. minima*	*B. ramanantsoai*	*B. dentata*	*B. karchei*	*B. peyrierasi*	*B. tuberculata*
N dorsolateral spines on body	10–12 (typically 11)	8–13 (typically indistinct)	11–12 (typically 11)	12–14 (typically 13)	11–12	14 (often indistinct/not countable)	4–11 (irregular)	10–11	13	10–11	9 (mostly not countable)
Pelvic spine	present	present (very small)	present	present	present	indistinct/absent	present	present	present	present	indistinct/present
Supraocular cone	absent	absent	absent	present/typically indistinct	absent	absent	absent or present	absent	present	absent	present
Supranasal cone	present	absent	present	present	present	ambiguous	present	present	present	present	present
Lateral spines on tail	present or absent	indistinct or absent	absent	present and distinct	absent	present or absent	absent	indistinct	present	present or absent	present or absent
Dorsal spines on tail	absent or indistinct	present or absent	absent	present or absent	absent	present or absent	absent	present and distinct	indistinct	absent	absent
N enlarged lateral head tubercles	1	1–2	1	3	1	1–2	0–2	2–3	1	1	1–2
Posterior crest	present	present or indistinct	present	present	present or indistinct	present or indistinct	absent or indistinct	present	present	present or indistinct	indistinct
Hemipenis	wide, small spine-like papillae on apex	very narrow, without ornamentation except some pustules on apex	wide, tubular, comb-like apical structure with 6 papillae	wide, two apex processes each with terminal spine	unknown	balloon-like without ornaments	balloon-like without ornaments	unknown	unknown	massive, bilobed, 4 spines per lobe	long, narrow, tubular, terminal crown-like structure

For original data see Supporting Information S1. Morphological data on *B. dentata* are based on photographs of A. Mori.

**Table 5:** Factor loadings of Principal Component Analyses.

Males	Factor 1	Factor 2	Factor 3
SVL	**−0.957045**	−0.076540	0.122373
TaL	−0.622996	**−0.738080**	0.234927
HW	**−0.941967**	0.117741	−0.167709
HH	**−0.964514**	0.085376	−0.155503
ED	**−0.735807**	0.579285	0.338150
FORL	**−0.942026**	−0.091742	−0.216903
Explained Variance (%)	75.8	15.3	4.7
Eigenvalue	4.55	0.92	0.28

Females	Factor 1	Factor 2	Factor 3
SVL	**−0.963446**	−0.081855	−0.035425
TaL	**−0.733371**	**−0.651325**	−0.067059
HW	**−0.925048**	0.226107	0.247520
HH	**−0.951288**	0.081203	0.239565
ED	**−0.835444**	0.420991	−0.314916
FORL	**−0.922966**	−0.088405	−0.119679
Explained Variance (%)	79.61	11.2	4.0
Eigenvalue	4.78	0.67	0.24

Analyses were performed separately for males and females based on all morphometric measurements of available specimens of the *B. minima* group (as in Tables 1–2 and [5]). Values over 0.7 in bold. Abbreviations as in Table 1.

Scatterplots of Factor 1 vs. Factor 2 in both males and females shows that the main divergence between species is along Factor 1, indicating consistent size differences. *B. minima*, *B. tuberculata* and *B. micra* are the smallest species, whereas *B. karchei* and *B. desperata* are the largest species. Factor 2, mainly influenced by tail length, clearly separates *B. micra* from the other species.

In order to translate these data into diagnostically relevant information, we performed univariate analyses of those variables that were identified as most relevant by PCA. For this purpose we normalized tail length (TaL), head height (HH), head width (HW) by dividing them by SVL. We chose this procedure, because the obtained ratio values can be directly used in species diagnoses. Data for males and females are summarized in Table 3, but due to missing data for females in several species, data were only visualized for males (Fig. 4c–d). The data confirm the differences in body size as summarized above, and indicate that indeed *B. micra* can be very clearly distinguished from all other species by its much shorter relative tail length (Fig. 4c). Some differences in relative tail length are also apparent among other species, but do not seem to be diagnostic, as there is great variation within species.

Values of HH/SVL and HW/SVL were correlated (Fig. 4d), suggesting that species and specimens with relatively wider heads also have taller heads. Again, several species can be diagnosed by a combination of relative head length and relative head height, in particular *B. micra* with relatively very tall and wide heads, and *B. minima* with the lowest relative head height and width.

ANCOVA analyses of the morphometric variables revealed significant differences among most pairs of species, largely congruent in separate analyses of male and female values (Table 6). In particular, *B. micra* differs in tail length from all other species whereas *B. minima* and *B. desperata* differ in head proportion (HH, HW, ED) from all or most other species.

**Table 6:** Summary of ANCOVA comparisons among species in the *B. minima* group.

	*B. micra*	*B. minima*	*B. tuberculata*	*B. tristis*	*B. confidens*	*B. peyrierasi*	*B. ramanantsoai*	*B. desperata*
***B. micra***	—	TAL, HW	TAL	TAL	TAL, FORL	SVL, TAL, HH, FORL	n.a.	SVL, TAL, HW, HH, ED, FORL
***B. minima***	TAL, HW, HH, ED	—	HW	HW	HW, HH, FORL	SVL, TAL, HW, HH, FORL	n.a.	SVL, TAL, HW, HH, ED, FORL
***B. tuberculata***	TAL, FORL	HW, HH, ED, FORL	—		HH, FORL	SVL, TAL, HW, HH, FORL	n.a.	SVL, HW, HH, ED, FORL
***B. tristis***	TAL	HW, HH, ED	FORL	—		TAL	n.a.	SVL, HW, HH, FORL
***B. confidens***	TAL	HW, HH, ED, FORL	ED, FORL		—	TAL	n.a.	SVL, HW, HH, FORL
***B. peyrierasi***	SVL, TAL, FORL	SVL, TAL, HW, HH, ED, FORL	TAL, FORL	TAL, HH, FORL	TAL	—	n.a.	SVL, HW, HH, ED, FORL
***B. ramanantsoai***	TAL	SVL, TAL, HW, HH, FORL	TAL, FORL	TAL	TAL	TAL	—	n.a.
***B. desperata***	SVL, TAL, HW, HH, FORL	SVL, TAL, HW, HH, ED, FORL	SVL, TAL, HW, HH, ED, FORL	SVL, TAL, HW, HH, FORL	SVL, HW, HH, FORL	SVL, HW, HH, ED, FORL	FORL	—

The table lists for separate analyses of males (above the diagonal) and females (below the diagonal) those morphometric variables that had significant differences in ANCOVAs (SVL used as covariable) with Scheffé's Post-Hoc analyses. SVL was compared among species without covariable. Only species included with at least 3 available specimens. Abbreviations as in Table 1.

**Figure 5:**
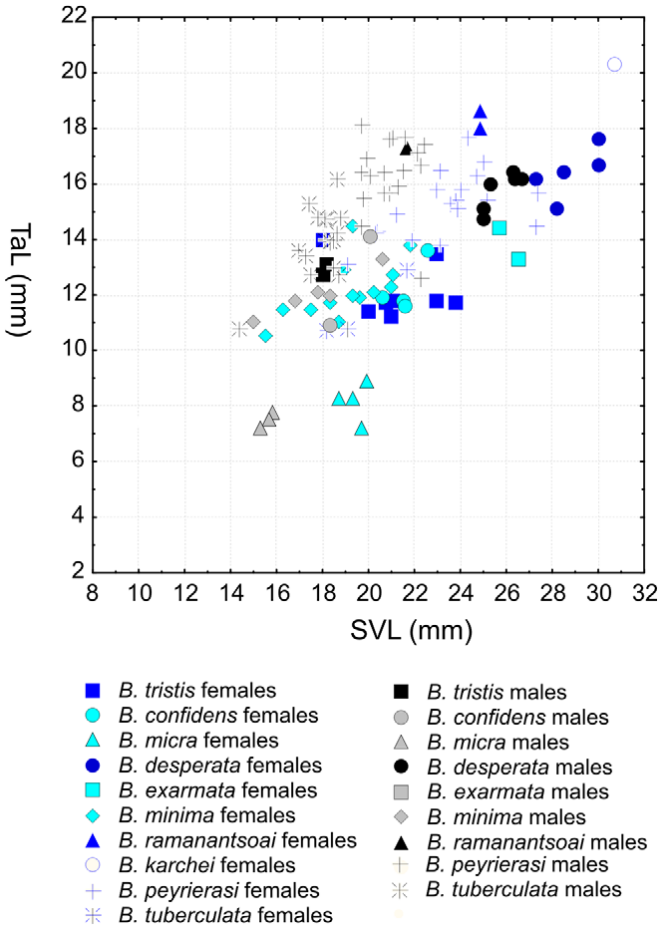
Morphometric differentiation among sexes in species of the *Brookesia minima* group. Scatterplot of tail length vs. snout-vent length in male and female specimens of the *Brookesia minima* group, showing larger body sizes in females and a weak trend towards relatively longer tails in males. Based on measurements in Tables 1 and 2, Supporting Information S1 and [5].

Sexual dimorphism in all studied species and populations pertain mainly to size, with females being distinctly larger than males, often without overlap (Fig. 5). Tail length, which in some other chameleon genera shows sexual dimorphism, with males having relatively longer tails than females, seems undifferentiated between sexes in the *B. minima* group. A slight trend of relatively longer tails in males is apparent in some species (Fig. 5) but could not be statistically verified due to a low sample size in many species.

Examination of several quantitative characters of external morphology previously used to distinguish species of *Brookesia* confirmed that most species in the *B. minima* group can be readily identified by a combination of morphological characters (Table 4; Supporting Information S1).

The constant presence of supraocular cones characterizes *B. tuberculata*, whereas such cones are absent in most other species and only visible in some individuals of *B. ramanantsoai*, *B. desperata*, and *B. karchei*. Supranasal cones are present in most species, but are absent in *B. confidens* and not clearly visible in *B. minima*. Most species have 1–2 enlarged lateral head tubercles, but *B. desperata* and some *B. dentata* have 3 such tubercles. Pelvic spines are present in most species, but are absent or indistinct in *B. minima*. Most species lack lateral spines on the tail, but in *B. desperata* these spines are very distinct. Several other characters, such as the number of dorsolateral spines on the body, are variable within species and show overlapping values among species. Still, in some cases this character can be informative for a diagnosis (Tables 3, 4).

### Divergence in genital morphology

Within the *Brookesia minima* group, hemipenes have been described for three species (*B. peyrierasi*, *B. ramanantsoai* and *B. tuberculata*) [5], [18], [36]. We herein provide data on genital morphology for the four newly described species, and for *B. minima* (see species accounts below). Hence, within the group, the genital morphology remains unknown only for *B. dentata*, *B. exarmata* and *B. karchei*.

**Figure 6:**
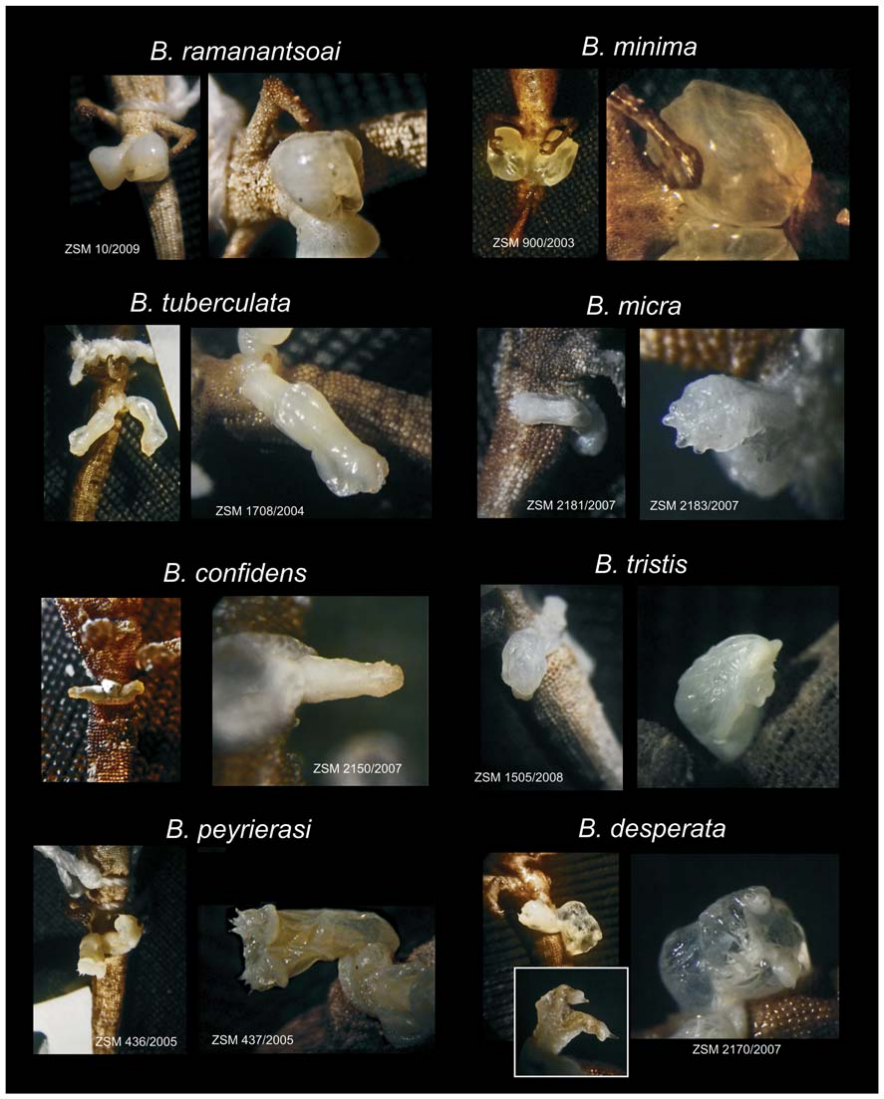
Hemipenes of species in the *Brookesia minima* group. The photos show for each species, a general view of the organs and a close-up. For *B. desperata*, the inset picture shows a non-turgid everted hemipenis where the two apex projections are very prominent. Note that also several other of the shown preparations are not fully turgid, especially in *B. ramanantsoai* and *B. micra*. In two other species (*B. confidens* and *B. tristis*) the shown hemipenes might not be fully everted.

In general, the hemipenes found in the *B. minima* group are remarkably dissimilar among species. Some structures found in other squamates, including other chameleons, are missing. For instance, in all species of the *B. minima* group, the hemipenes are devoid of spines, papillae, paryphasmata or calyces on the pedicel and truncus (Fig. 6). The sulcus spermaticus is only poorly visible in many of the examined organs, and because of differences in turgidity, we are not confident regarding the true state and shape of the sulcus in all specimens. We have therefore excluded this character from the hemipenis descriptions provided below.

In several species, there is quite extensive material available. For instance, in *B. peyrierasi*, *B. tuberculata*, and *B. desperata*, we have examined hemipenes of five or more specimens. Despite great differences in the state and method of fixation and preservation and the degree of hemipenis eversion, the species-specific characters of these three species were visible in all specimens and each of them was identifiable to species on the basis of their genitalia alone. This bolsters our confidence that our observations in less well-represented species accurately reflect their true morphological variability, and that we have correctly identified species-specific characteristics as summarized in the following.

Within the *B. minima* group, two species (*B. minima* and *B. ramanantsoai*) can be recognized by their large and balloon-like hemipenes without any recognizable ornamentation (Fig. 6; Supporting Information S1). Very wide and, when turgid, somewhat balloon-like organs are also found in *B. desperata* and *B. tristis*, but these species differ from *B. minima* and *B. ramanantsoai* by the presence of large or small spines, respectively, and a clearly cylindrical rather than globular form. The hemipenes of *B. desperata* uniquely possess two tubular processes on the apex, especially visible in non-turgid organs, each with a large and distinct terminal spine. The hemipenes of *B. peyrierasi* have a bilobed apex, with four smaller spines on each lobe. *Brookesia tuberculata* has long, narrow, tubular hemipenes without ornamentation except a very small crown-like structure on the apex. Our data for *B. tristis* and *B. confidens* are based on single or few specimens only. However, it seems clear that *B. tristis* has a relatively wide truncus with some tiny spine-like papillae in the apex region, without showing the massive truncus and clear bilobed apex of *B. peyrierasi*, and *B. confidens* apparently has a very long and narrow organ without ornamentation.

### New species descriptions

The data presented above provide overwhelming evidence for a concordant differentiation of all described species of the *B. minima* group. The four newly discovered populations from Montagne des Français, Ankarana, Nosy Hara and Forêt d'Ambre show clear differences in nuclear and mitochondrial DNA sequences, morphometry, and qualitative characters of external and genital morphology. The degree of genetic differentiation is furthermore extremely high and indicates very old lineage divergence [14]. It seems clear that all these forms are distinct evolutionary lineages warranting species status, and we therefore provide herein formal taxonomic descriptions of these four new species. The diagnoses of the new species are organized sequentially, i.e., they do not provide distinctions from those new species described in subsequent sections. A full comparison of most diagnostic characters is given in Tables 3, 4. A tentative determination key based on morphological characters is included in Supporting Information S1.

***Brookesia tristis*** **sp. n.** ZooBank LSID: urn:lsid:zoobank.org:act:56F36D4D-1F94-49C4-AC30-6E894B6AA998

*Remark*.—This species has been considered before as *Brookesia* sp. “Montagne des Francais” [7] and as *Brookesia* sp. nov. [37].

*Holotype*.— ZSM 1704/2004 (no field number), adult male (hemipenes everted), collected at Montagne des Français, 12°19′S, 49°20′E, ca. 150 m a.s.l., Antsiranana Province, northern Madagascar, on 23 February 2004 by F. Glaw, M. Puente, R. D. Randrianiaina and guides of the hotel “King's Lodge”.

*Paratypes*.— UADBA uncatalogued (FGZC 477–478), ZSM 357/2004 (FGZC 654) [not examined morphologically], and ZSM 876/2010 [no field number, not examined morphologically], adult males, ZSM 354/2004 (FGZC 651), adult female, same data as holotype; ZSM 1705/2004–1707/2004 (no field numbers), three adult females, most likely same data as holotype. UADBA uncataloged (FGZC 1187, 1189, 1191); ZSM 2146–2149/2007 (FGZC 1188, 1190, 1192, 1193), adult females, all collected at Montagne des Français, near remains of French Fort, 12°19′S, 49°20′E, 250–300 m a.s.l., Antsiranana Province, northern Madagascar, on 27 February 2007 by P. Bora, H. Enting, F. Glaw, A. Knoll and J. Köhler; ZSM 2018/2008 (FGZC 1733 = MgF 061) and UADBA uncatalogued (FGZC 1734 = MgF 061), collected at Montagne des Français, canyon 2 km [air distance] W of Andavakoera, 12°19,838′S, 49°20,941′E, 250 m a.s.l., on 24 January 2006 by E. Randriamalala; UADBA uncatalogued (FGZC 1657), female, ZSM 1505/2008 (FGZC 1656), adult male (hemipenes everted), both collected ca. 1.5 km southwest of Andavakoera (“Frontier base camp”), 12°19′59.2″S, 49°21′20.6″E; 140 m a.s.l., Montagne des Français, Antsiranana Province, northern Madagascar, on 16 February 2008 by M. Franzen, F. Glaw, J. Köhler and Z.T. Nagy.

*Diagnosis*.— A member of the *Brookesia minima* group based on small body size (SVL <24 mm) and molecular phylogenetic relationships. *Brookesia tristis* is distinguished from other members of the group as follows: from *B. dentata* by probably smaller adult body size (no measurements of male *B. dentata* available), and presence (in most specimens) of lateral spines on the tail (vs. absence); from *B. exarmata* by presence (in most specimens) of lateral spines on the tail (vs. absence); from *B. karchei* by probably smaller adult body size (female SVL 18.0–23.8 mm vs. 30.7 mm; no measurements of clearly identified males available for *B. karchei*), and absence of a supraocular cone (vs. presence); from *B. minima* by the presence of a supranasal cone, a pelvic spine, and (in most specimens) lateral spines on the tail (vs. absence), and hemipenis with small apical spine-like papillae (vs. balloon-like without ornaments); from *B. peyrierasi* by smaller adult body size (male SVL 18.0–18.2 mm vs. 19.7–22.4 mm), presence (in most specimens) of lateral spines on the tail (vs. absence), and by a hemipenis with small apical spine-like papillae (vs. bilobed hemipenis with four large spines per lobe); from *B. ramanantsoai* by smaller adult body size (male SVL 18.0–18.2 mm vs. 21.7 mm), absence of a supraocular cone (vs. presence in some specimens), presence (in most specimens) of lateral spines on the tail (vs. absence), and hemipenis with small apical spine-like papillae (vs. hemipenis balloon-like without ornaments); and from *B. tuberculata* by the absence of a supraocular cone (vs. presence), presence (in most specimens) of lateral spines on the tail (vs. absence), and hemipenis with small apical spine-like papillae (vs. hemipenis with apical crown-like structure). For a distinction from *B. confidens*, *B. desperata*, and *B. micra*, described below, see the diagnoses of these species. Referencing a fragment of the 16S rRNA gene, *B. tristis* shows an uncorrected pairwise divergence of 6.4% to its sister species *B. desperata*, and divergences >6.9% to all other species of the *B. minima* group.

**Figure 7:**
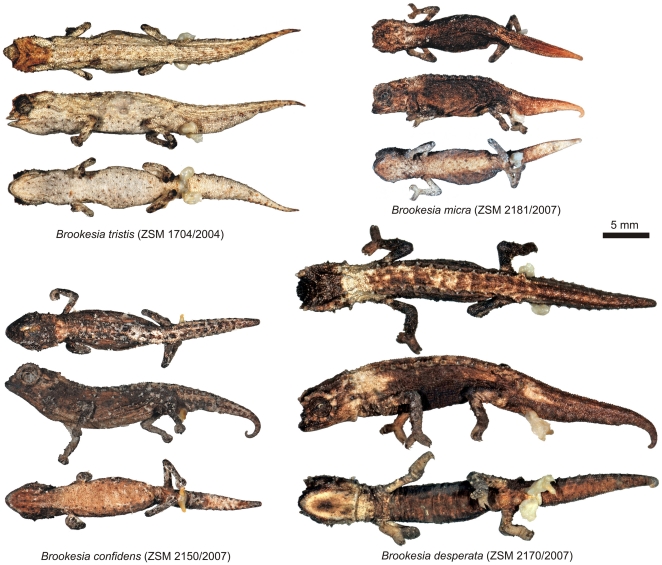
Dorsal, lateral and ventral views of preserved male holotypes of newly described species. Scale bar equals 5 mm.

*Description of holotype*.— Adult male in good state of preservation (Fig. 7; Supporting Information S1). Both hemipenes everted. Measurements in Table 1. Head with lateral crest starting at median level at the posterior edge of eye, prominent orbital crests, and a crest at the posterior edge of the head, that form a weakly developed dorsal helmet; a pair of curved parasagittal crests that start above the eyes and begin to converge before terminating at the posterior crest; between the parasagittal crests there is a second pair of parallel longitudinal crests; three pointed tubercles on each side of posterior helmet crest, one at termination point of lateral crest, one at termination point of parasagittal crest, and one between parasagittal and lateral crests; one pointed tubercle on lateral surface of head, below lateral crest in temporal region; orbital crest denticulated; no supraocular cone recognizable; supranasal cone does not project beyond snout tip; head longer (5.1 mm) than wide; chin and throat without longitudinal rows of slightly enlarged tubercles. Dorsal surface of body without a vertebral ridge or keel; 11 dorsolateral pointed tubercles form a complete longitudinal line on the body; 10th dorsolateral tubercle least pointed, small; most posterior (11th) pointed dorsolateral tubercle being largest, above insertion point of hindlimb, very slightly projecting backwards; pointed dorsolateral tubercles almost equally spaced, 3rd to 6th tubercle slightly larger than others, pointing out almost perpendicularly from body; slightly enlarged, rounded tubercles form curved transversal crests on either side of the vertebral line between 1st and 10th dorsolateral pointed tubercles; dorsal surface of tail with slightly enlarged rounded tubercles forming separated rounded crests that continue from tailbase to two-thirds down the tail; no well-defined dorsal pelvic shield in sacral area; lateral surface of body with evenly spaced enlarged rounded tubercles, mainly arranged in four longitudinal rows; venter without enlarged tubercles; scattered, soft-pointed tubercles on limbs; no pointed tubercles around cloaca; longitudinal row of slightly enlarged pointed tubercles lateral on tail, forming a longitudinal row from tail base to tail tip; no enlarged tubercles on ventral surfaces of tail. In life, colouration of head, body, limbs and tail brown to beige. Faint brown bars radiate from eye to lateral surfaces of head; chin and throat brown; ventral surfaces of body and tail pale brown. After almost seven years in ethanol, all surfaces pale grey to beige, with most pointed tubercles being brown; upper surface of head pale brown.

*Variation*.— For morphological measurements and proportions see Table 1 and Supporting Information S1. In preservative, most paratypes are dorsally darker than the holotype, ranging from brown to dark brown. Their ventral surface is greyish except for the region of the chest, which shows brown mottling. In life several specimens got a light grey vertebral stripe when kept together with conspecifics in a bag, suggesting that this might be a stress colouration. The life colouration of juveniles generally resembles that of adults. The tail base of the females is distinctly less thickened than in the males. In life, two adult specimens had a weight of 0.18 g and 0.206 g, respectively.

*Genital morphology*.— Only two specimens with everted hemipenes were available for examination: ZSM 1505/2008 and ZSM 1704/2004 (holotype). In both specimens, one hemipenis is clearly incompletely everted whereas the second one appears fully everted, although this cannot be stated with complete certainty. In ZSM 1505/2008, hemipenis length is 2.5 mm and maximum hemipenis width is 1.7 mm when fully turgid. The hemipenis is an irregular tubular structure without any ornaments, which in fully turgid state is somewhat balloon-shaped, widest at the central part of the truncus and more narrow at the pedicel and apex. Near the apex, two very small lobe-like structures are visible, corresponding to attachments of the retractor muscle, and a few very small spine-like papillae. However, it is not clear whether the hemipenis could possibly become further everted and then may become more strongly bilobed terminally, with a more prominent exposure of the spine-like papillae. There are no ornaments on pedicel and truncus, and no recognizable ornamental structures on apex (although apex might not be fully everted; Fig. 6).

*Etymology*.— The species epithet is an adjective derived from the Latin “tristis” meaning “doleful”, “sad”, “sorrowful”, and refers to the fact that the entire known range of this species (Montagne des Français) suffers from severe deforestation and habitat destruction [37] despite recently being declared as a nature reserve.

*Distribution*.— Only known from the Montagne des Français limestone massif.

*Natural History*.— Most individuals of *B. tristis* were found roosting at night on small branches about 5–50 cm above the leaf litter within a limestone massif with deciduous dry forest. By far most specimens were found in a few small areas where the species was abundant, whereas it was rare in other, apparently similar habitats. One of the females laid two large eggs (5.9×3.7 mm resp. 5.8×3.5 mm diameter) in February. The two juveniles hatched 64 and 69 days after egg deposition at temperatures between 20–26°C. They measured 14 mm in total length. Eight days after hatching the weight of the older juvenile was 0.03 g.

***Brookesia confidens*** **sp. n.** ZooBank LSID: urn:lsid:zoobank.org:act:CB2A9146-0161-42B7-A145-6DED579F1F21

*Remark*.—This species has been considered before as conspecific with *Brookesia* sp. “Montagne des Francais” [7].

*Holotype*.— ZSM 2150/2007 (FGZC 1196), adult male (hemipenes incompletely everted), collected on trail to the “Petit Tsingy and Grotte des Chauves-Souries”, 12°57′25″S, 49°07′06″E, 90 m a.s.l., Ankarana National Park, Antsiranana Province, northern Madagascar, on 1 March 2007 by P. Bora, H. Enting, F. Glaw, A. Knoll, and J. Köhler.

*Paratypes*.— UADBA uncatalogued (FGZC 1194–1195), two specimens; ZSM 2151/2007 (FGZC 1197), probably male, ZSM 2152/2007 (FGZC 1198), adult female, ZSM 2153/2007 (FGZC 1199), adult female, all with same data as holotype; UADBA uncatalogued (FGZC 1608), UADBA uncatalogued (FGZC 1610), ZSM 1511/2008 (FGZC 1607), adult female, ZSM 1512/2008 (FGZC 1609), adult male, all collected at “Petit Tsingy”, Ankarana National Park, Antsiranana Province, northern Madagascar, on 12 February 2008 by N. D'Cruze, M. Franzen, F. Glaw and J. Köhler.

*Diagnosis*.— A member of the *Brookesia minima* group based on small body size (SVL<23 mm) and molecular phylogenetic relationships. *Brookesia confidens* is distinguished from other members of the group as follows: from *B. dentata* by probably smaller adult body size (no measurements of male *B. dentata* available), and absence of a supranasal cone (vs. presence); from *B. exarmata* by the absence of a supranasal cone (vs. presence); from *B. karchei* by a smaller adult body size (female SVL 20.6–22.6 mm vs. 30.7 mm; no measurements of clearly identified males available for *B. karchei*), and absence of a supranasal cone (vs. presence); from *B. minima* by a very narrow hemipenis (vs. balloon-like); from *B. peyrierasi* by generally smaller adult body size(male SVL 18.3–20.1 mm vs. 19.7–22.4 mm), the absence of a supranasal cone (vs. presence), absence of a supraocular cone (vs. presence), and very narrow ornamentless hemipenis (vs. massive, bilobed hemipenis with four spines per lobe); from *B. ramanantsoai* by a smaller adult body size (male SVL 18.3–20.1 mm vs. 21.7 mm), absence of a supranasal cone (vs. presence), absence of a supraocular cone (vs. presence in some specimens), and hemipenis very narrow (vs. balloon-like); from *B. tuberculata* by the absence of a supranasal cone (vs. presence), absence of a supraocular cone (vs. presence), and hemipenis very narrow with pustules on apex (vs. wider, with crown-like apical structure). The new species is most similar to *B. tristis* but differs from this species by indistinct and short parasagittal crests (vs. distinct) and by 13 dorsolateral pointed tubercles (vs. 11), and ornamentless apical region of hemipenis (vs. spine-like papillae on apex). For a distinction from *B. desperata* and *B. micra*, described below, see the diagnoses of these species. Referencing a fragment of the 16S rRNA gene, *B. confidens* shows an uncorrected pairwise divergence of 6.7% to its sister species *B. tuberculata*, and divergences >9% to all other species of the *B. minima* group.

*Description of holotype*.— Adult male in good state of preservation (Fig. 7; Supporting Information S1). Both hemipenes everted. Measurements in Table 1. Head with lateral crest starting at median level at the posterior edge of eye; prominent orbital crests with distinctly enlarged pointed tubercle at posterior level slightly below lateral crest, and a crest at the posterior edge of the head, that form a weakly developed dorsal helmet; a pair of very short straight parasagittal crests that transform posteriorly into a broad area of elevated scales which almost cover the entire posterior head surface; three pointed tubercles on each side of posterior helmet crest, a prominent one at termination point of lateral crest, an even larger one at the imaginary termination point of parasagittal crest, and a weakly developed one between parasagittal and lateral crests; one pointed tubercle on lateral surface of head, below lateral crest in temporal region; orbital crest denticulated; no supraocular cone recognizable; supranasal cone not clearly recognizable; head longer (5.3 mm) than wide; chin and throat without longitudinal rows of slightly enlarged tubercles. Dorsal surface of body without a vertebral ridge or keel; 13 dorsolateral pointed tubercles form a complete longitudinal line on the body; most posterior (13th) pointed dorsolateral tubercle being largest, above insertion point of hindlimb; pointed dorsolateral tubercles almost equally spaced, pointing out almost perpendicularly from body; slightly enlarged, pointed tubercles form curved transversal crests on either side of the vertebral line between 2nd and 10th dorsolateral pointed tubercles; slightly enlarged tubercles on dorsal surface of tail, without any reconizable pattern; no well-defined dorsal pelvic shield in sacral area; few irregularly scattered enlarged rounded ubercles on the lateral surface of body; venter without enlarged tubercles; scattered, soft-pointed tubercles on limbs; no pointed tubercles around cloaca; longitudinal row of slightly enlarged pointed tubercles lateral on tail, forming a longitudinal row from tail base to two-thirds of tail length; no enlarged tubercles on ventral surfaces of tail. After almost four years in ethanol, all surfaces pale grey to beige, with most pointed tubercles being brown; upper surface of head pale brown.

*Variation*.— For morphological measurements and proportions see Table 1 and Supporting Information S1. In ZSM 2153/2007, the ground colour is dark brown with a whitish vertebral stripe that starts at posterior half of body and extends onto the tail which is entirely beige with scattered brownish spots. The posterior dorsal surface of head is grey. The body of ZSM 1512/2008 is light grey whereas the head is brown with a greyish transverse line between the nostrils. The tail base of the females is less thickened than in the males. In life, all individuals with dorsal colouration of head, dorsum and tail light grey or pale beige, lateral parts of the body brown, with indistinct greyish marbling lateroventrally and with few small dark brown spots around the flank tubercles; limbs almost uniformly dark brown. This pattern is likely a stress colouration.

*Genital morphology*.— For this species, everted hemipenes are available only for the holotype (ZSM 2150/2007). On both sides, the organs are single tube-like structures of low diameter which decreases from the apex to the distal part of the truncus. The left hemipenis is not fully everted whereas the one on the right side (length 1.3 mm; width at the pedicel 0.6 mm, width at the apex 0.4 mm) might be fully everted, but this cannot be verified without examination of further material. There are no ornaments on the pedicel and truncus. The sulcus spermaticus is not recognizable on most of the truncus but becomes more distinct in the apical region. The apical region is characterized by a slight increase in diameter but not bilobed, and is covered by relatively large but poorly defined pustular papillae (Fig. 6). The hemipenis is devoid of any further prominent structures such as the spiny apical papillae of *B. peyrierasi* (which are visible also in incompletely everted organs).

*Etymology*.— The species epithet is an adjective derived from the Latin “confidens” meaning “confident”, “trusting”. The known range of the species is supposedly a well protected nature reserve with apparently limited habitat destruction. Furthermore, this area might benefit from natural protection by the tsingy limestone formations which are difficult to access, thus giving hope for the species' survival.

*Distribution*.— Only known from a single locality within Ankarana National Park.

*Natural History*.— Most individuals were found roosting at night on thin branches about 5–20 cm above the leaf litter in deciduous dry forest close to a small forest trail within a small area, surrounded by tsingy outcrops. At this locality, the species was relatively abundant, whereas it was not found at similar localities nearby, suggesting a patchy distribution and a preference for certain microhabitats. This hypothesis is also supported by the fact that earlier herpetological surveys in Ankarana [38], [39], did not record any species of the *Brookesia minima* group.

When stressed, individuals can quickly change colour and display a broad pale vertebral stripe contrasting with the darker flanks.

***Brookesia micra*** **sp. n.** ZooBank LSID: urn:lsid:zoobank.org:act:D1A239D6-93E8-4C34-A428-F79A2C8B6405

*Remark*.—This species has been considered before as *Brookesia* sp. “Nosy Hara” [7].

*Holotype*.— ZSM 2181/2007 (FGZC 1271), adult male (hemipenes everted), collected on Nosy Hara island, 12°14′40″S, 49°00′30″E, ca. 10–20 m a.s.l., Antsiranana Province, northern Madagascar, on 7 March 2007 by H. Enting, F. Glaw and J. Köhler.

*Paratypes*.— ZSM 2180/2007 (FGZC 1270), ZSM 2182/2007 (FGZC 1275), juveniles, ZSM 2183/2007 (FGZC 1278), ZSM 2185/2007 (FGZC 1280), adult males (hemipenes everted), ZSM 2184/2007 (FGZC 1279), ZSM 2186–2187/2007 (FGZC 1281–1282), adult females, all with same data as holotype; UADBA uncatalogued (FGZC 1830), male, ZSM 1507/2008 (FGZC 1831), male (not examined morphologically), UADBA uncatalogued (FGZC 1833), female, ZSM 1509/2008 (FGZC 1834), adult female, ZSM 1510/2008 (FGZC 1832), juvenile, all collected at small stream on Nosy Hara, 12°14′59″S, 49°00′28″E, 12 m a.s.l., Antsiranana Province, northern Madagascar, on 22 February 2008 by F. Glaw and J. Köhler.

*Diagnosis*.— A member of the *Brookesia minima* group based on small body size (SVL<20 mm) and molecular phylogenetic relationships. *Brookesia micra* is distinguished from all other members of the group by a shorter relative tail length (tail length/SVL 0.37–0.49 versus 0.49–0.92), and by orange coloured tails in life in adults (vs. inconspicuous brownish colour). In addition, from *B. confidens* by a smaller adult male size (SVL 15.1–15.3 mm vs. 18.3–20.1 mm), supranasal cone present (vs. absent), and hemipenis with comb-like apical structure (vs. narrow without ornaments); from *B. dentata* by probably smaller adult body size (no measurements of male *B. dentata* available); from *B. exarmata* by smaller adult body size (female SVL 18.7–19.9 vs. 25.7–26.5, no male measurements available for *B. exarmata*); from *B. karchei* by smaller size (female SVL 18.7–19.9 vs. 30.7, no male measurements available for *B. karchei*); supraocular cone absent (vs. present); from *B. minima* by presence of a pelvic spine (vs. absent or indistinct pelvic spine), and hemipenis with comb-like apical structure (vs. balloon-like without ornaments); from *B. peyrierasi* by a smaller adult male size (SVL 15.1–15.3 mm vs. 19.1–27.4 mm), and hemipenis with comb-like apical structure (vs. bilobed with four spines on each lobe); from *B. ramanantsoai* by a smaller adult male size (SVL 15.1–15.3 mm vs. 21.7 mm), supraocular cone absent (vs. present in some specimens), and hemipenis with comb-like apical structure (vs. baloon-like without ornaments); from *B. tristis* by a smaller adult male size (SVL 15.1–15.3 mm vs. 18.0–18.2 mm), and hemipenis with comb-like apical structure (vs. small papillae on apex not arranged comb-like); and from *B. tuberculata* by supraocular cone absent (vs. present), and hemipenis with comb-like apical structure (vs. crown-like structure). For a distinction from *B. desperata*, described below, see the diagnosis of this species. Referencing a fragment of the 16S rRNA gene, *B. micra* shows an uncorrected pairwise divergence of 6.8% to its sister clade (*B. tristis+B. desperata*), and divergences >7.2% to all other species of the *B. minima* group.

*Description of holotype*.— Adult male in good state of preservation (Fig. 7; Supporting Information S1). Both hemipenes everted. Measurements in Table 2. Head with lateral crest starting at median level at the posterior edge of eye; prominent orbital crests; a weak crest at the posterior edge of the head, that forms a weakly developed dorsal helmet; a pair of low indistinct parasagittal crests slightly converging before terminating at the lateral crest; three similar-sized pointed tubercles on each side of posterior crest, one at termination point of lateral crest, one at termination point of parasagittal crest, and a one between parasagittal and lateral crests; one pointed tubercle on lateral surface of head, below lateral crest in temporal region; orbital crest denticulated; no supraocular cone recognizable; supranasal cone small; head longer (4.5 mm) than wide; chin and throat without longitudinal rows of slightly enlarged tubercles. Dorsal surface of body without a vertebral ridge or keel; 11 dorsolateral pointed tubercles form a complete longitudinal line on the body; most posterior (11th) pointed dorsolateral tubercle being largest, above insertion point of hindlimb; pointed dorsolateral tubercles almost equally spaced, pointing out almost perpendicularly from body; slightly enlarged, rounded tubercles form curved transversal crests on either side of the vertebral line between 1st and 10th dorsolateral pointed tubercles; slightly enlarged scales forming four parallel longitudinal rows on dorsal surface of tail; no well-defined dorsal pelvic shield in sacral area; scattered enlarged rounded tubercles on the lateral surface of body; venter without enlarged tubercles; no pointed tubercles around cloaca; no enlarged tubercles on lateral or ventral surfaces of tail. After almost four years in ethanol, back, flanks, dorsal surfaces of limbs and anterior part of tail uniformly dark grey-brown; posterior part of tail beige; neck grey; throat and venter light grey.

*Variation*.— For morphological measurements and proportions see Table 2 and Supporting Information S1. In preservative, ZSM 2185/2007 and 2188/2007 have a greyish dorsal surface of head and neck and a slightly lighter vertebral region. The throat is uniformly dark brown (ZSM 2187/2007 and 2184/2007) or beige with brown spots (ZSM 2185/2007, 2188/2007, and 1509/2008). The tail base of the males is only slightly more thickened than in the females. In life, most individuals with dorsal colouration of head, dorsum and tail light grey, lateral parts of the tail more yellowish becoming orange posterior to the tail base. Lateral parts of the body brown, with few dark brown spots; limbs almost uniformly dark brown. This pattern is likely to refer to a stress colouration. When unstressed, most parts of the body dark brown, except a beige area on the head anterior to the eyes. Tail base dark brown, orange in the middle and yellowish posteriorly. Colour and pattern of juveniles resemble those of adults and they can also show the stress colouration, but tails are slightly less colourful than in adults and usually do not show bright orange colour.

*Genital morphology*.— Everted hemipenes of this species are available for three specimens (holotype ZSM 2181/2007, paratypes ZSM 2183/2007, ZSM 2185/2007). Each hemipenis is an elongated, relatively wide tubular structure. Apex and truncus are devoid of any ornaments. In the holotype, hemipenis length is 2.4 mm and maximum hemipenis width is 0.9 mm. When fully everted, the most characteristic feature is the apex which is a flat surface distally forming a symmetrical comb of six large, rounded papillae, of which the two inner ones are largest, followed by the two intermediate ones, whereas the outer ones are merely two slightly elevated knobs, recognizable only in fully everted organs. In general, this comb of papillae is not visible in hemipenes that are not fully everted, different from the spines of, e.g., *B. peyrierasi* that typically are also visible in incompletely everted organs. All three examined specimens have one hemipenis fully everted, the second one only partially everted. The organs are largely transparent, and the central retractor muscles can be easily seen through the outer hemipenial integument (Fig. 6).

*Etymology*.— The species epithet is a latinized derivation from the Greek word “μικρός” (mikros), meaning “tiny” or “small” and referring to the extremely diminutive body size. It is used as an invariable noun in apposition.

**Figure 8:**
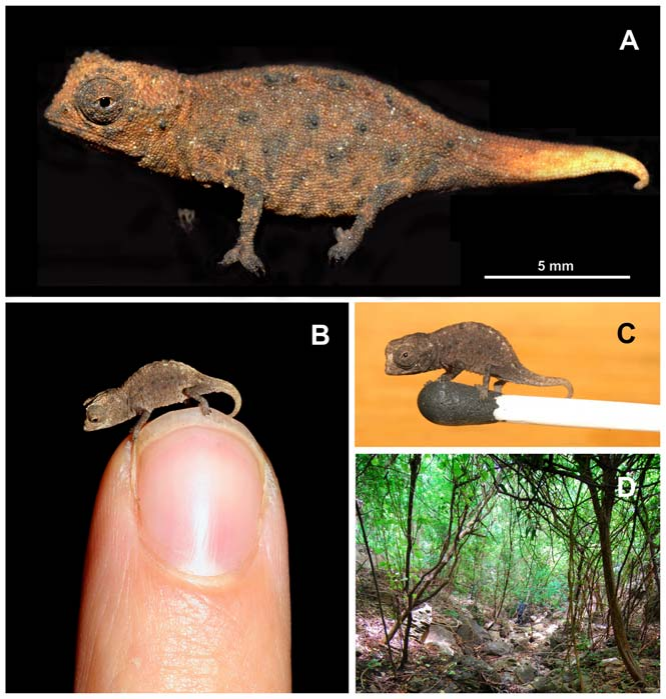
*Brookesia micra* sp. n. from Nosy Hara, northern Madagascar. (A) adult male on black background, showing orange tail colouration; (B) juvenile on finger tip; (C) juvenile on head of a match; (D) habitat along a small creek on western flank of Nosy Hara, where part of the type series was collected.

*Distribution*.— Only known from two sites (see localities in type series) on the small island of Nosy Hara, northern Madagascar (e.g. Fig. 8D). Remarkably, no *Brookesia* species was recorded during intensive herpetological surveys of Nosy Hara, nearby islands and the adjacent mainland [40], suggesting that *B. micra* might be difficult to record.

*Natural History*.— *B. micra* was found during the day active on the ground in a mosaic of eroded limestone boulders and dry forest leaf litter, and at night roosting on branches in very low vegetation (ca. 5–10 cm above the ground). In contrast to *B. tristis* and *B. confidens* its occurrence was not remarkably patchy.

### *Brookesia desperata* sp. n

ZooBank LSID: urn:lsid:zoobank.org:act:C62B456C-CDA8-4DDE-AE0A-66DF43C57F7E

*Remark*.—This species has been considered before as *Brookesia* sp. aff. *karchei* “Ambre” [7] and as *Brookesia* sp. nov. [41].

*Holotype*.— ZSM 2170/2007 (FGZC 1250), adult male (hemipenes everted), collected at Forêt d'Ambre Special Reserve, ca. 5 km southwest of Sakaramy village, 12°28′00″S, 49°13′37″ E, 470 m a.s.l., Antsiranana Province, northern Madagascar, on 12 March 2007 by F. Glaw, J. Köhler and A. Razafimanantsoa.

*Paratypes*.— ZSM 2171/2007 (FGZC 1251), ZSM 2175/2007 (FGZC 1258), ZSM 2176/2007 (FGZC 1260), ZSM 2177/2007 (FGZC 1263), ZSM 2179/2007 (FGZC 1269), adult males (all with everted hemipenes), ZSM 2172–2174/2007 (FGZC 1252–1254), ZSM 2178/2007(FGZC 1265), adult females, all with same data as holotype; ZSM 1506/2008 (FGZC 1880, female), and UADBA uncatalogued: FGZC 1700, FGZC 1879 (male), FGZC 3111–3112, collected at Forêt d'Ambre Special Reserve, ca. 5 km southwest of Sakaramy village, 12°28′S, 49°13′E, 550 m a.s.l., Antsiranana Province, northern Madagascar, on 27 February 2008 by N. D'Cruze, F. Glaw and J. Köhler; ZSM 791/2009 (ZCMV 13040), adult male (not examined morphologically), collected at Forêt d'Ambre Special Reserve, 12°28′18.0″S, 49°13′56″E, 438 m a.s.l., Antsiranana Province, northern Madagascar, on 16 November 2009 by A. Crottini, S. Hauswaldt, A. Lima, F. M. Ratsoavina and E. Rajeriarison.

*Diagnosis*.— A member of the *Brookesia minima* group based on small body size (SVL 25–30 mm) and molecular phylogenetic relationships. *Brookesia desperata* is distinguished from all other species in the group by the presence of three enlarged tubercles on lateral head surface (versus 0–2). In addition it differs as follows: from *B. confidens* by a larger adult body size (male SVL 25.0–26.7 vs. 18.3–20.1 mm), supranasal cone present (vs. absent), and hemipenis with two apical processes each with a distinct spine (vs. narrow hemipenis without apical ornaments); from *B. dentata* by presence of well-developed lateral spines on the tail (vs. absence); from *B. exarmata* by a larger adult body size (female SVL 27.3–30.0 mm vs. 25.7–26.5 mm), and presence of well-developed lateral spines on the tail (vs. absence); from *B. micra* by a larger adult body size (male SVL 25.0–26.7 vs. 15.3–15.8 mm), presence of well-developed lateral spines on the tail (vs. absence), and hemipenis with two apical processes each with a distinct spine (vs. comb-like arranged papillae on apex); from *B. minima* by a larger adult body size (male SVL 25.0–26.7 vs. 15.0–20.6 mm), presence of well-developed lateral spines on the tail (vs. indistinct), pelvic spine present (vs. absent or indistinct), and hemipenis with two apical processes each with a distinct spine (vs. balloon-like hemipenis without ornaments); from *B. peyrierasi* by a larger adult body size (male SVL 25.0–26.7 vs. 19.7–22.4 mm), presence of well-developed lateral spines on the tail (vs. indistinct), and hemipenis with two apical processes each with a distinct spine (vs. four spines on each lobe); from *B. ramanantsoai* by presence of well-developed lateral spines on the tail (vs. absence), and hemipenis with two apical processes each with a distinct spine (vs. balloon-like hemipenis without ornaments); from *B. tristis* by a larger adult body size (male SVL 25.0–26.7 vs. 18.0–18.2 mm), and hemipenis with two apical processes each with a distinct spine (vs. small spine-like papillae on apex); and from *B. tuberculata* by a larger adult body size (male SVL 25.0–26.7 vs. 14.4–18.8 mm), presence of well-developed lateral spines on the tail (vs. absence), and hemipenis with two apical processes with a distinct spine (vs. a single crown-like structure on apex).

**Figure 9:**
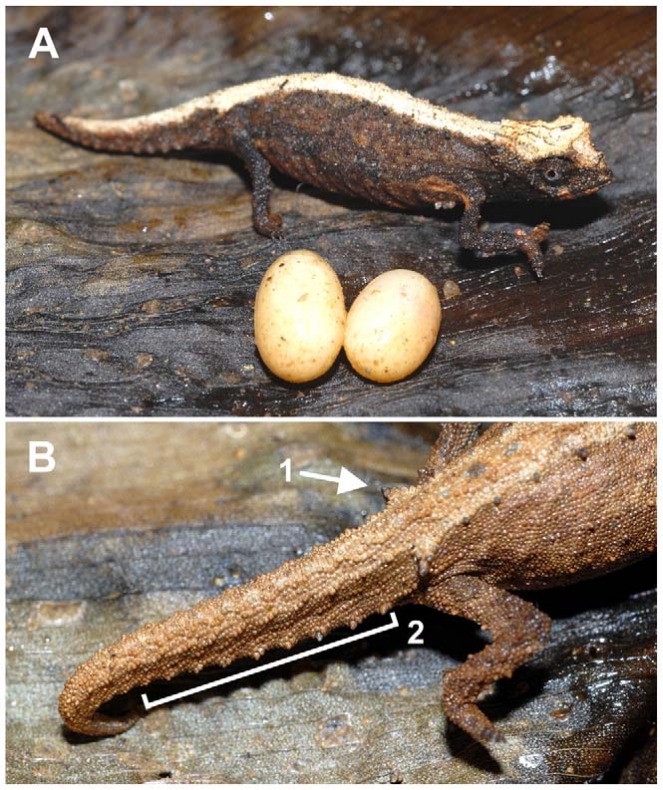
Life history and morphology of *Brookesia desperata*. (A) Female (displaying stress colouration) with two recently laid eggs. (B) Figure showing well-developed pelvic spine (1) and lateral spines on tail (2).

*B. desperata* is most similar to *B. karchei* in body size, number of dorsolateral pointed tubercles (12–13) and distinct lateral tubercles on tail (Fig. 9B). However, *B. karchei* differs from the new species by more pronounced supraocular and supranasal cones, more prominent and spiny posterior crest and only one enlarged tubercle at lateral side of head (three in *B. desperata*).

Referencing a fragment of the 16S rRNA gene, *B. desperata* shows an uncorrected pairwise divergence of 6.4% to its sister species *B. tristis*, and divergences >6.6% to all other species of the *B. minima* group.

*Description of holotype*.— Adult male in good state of preservation (Fig. 7; Supporting Information S1). Both hemipenes everted. Measurements in Table 2. Head with low lateral crest starting at median level at the posterior edge of eye; prominent orbital crests with distinctly developed supraocular cone directed anteriorly, and a crest at the posterior edge of the head, that form a weakly developed dorsal helmet; a pair of slightly curved parasagittal crests that start above the eyes and begin to converge slightly before terminating at the posterior crest; between the parasagittal crests there is a second pair of short parallel longitudinal crests; three pointed tubercles on each side of posterior helmet crest, one at termination point of lateral crest, one at termination point of parasagittal crest, and one between parasagittal and lateral crests; three pointed tubercles on lateral surface of head, one below lateral crest in temporal region, one at posterior edge of orbital crest below lateral crest, and one between lower edge of eye slightly anterior to angle of jaws; orbital crest with few enlarged conical tubercles; no supraocular cone recognizable; supranasal cone does not project beyond snout tip; head longer (6.4 mm) than wide; chin and throat without longitudinal rows of slightly enlarged tubercles. Dorsal surface of body without a vertebral ridge or keel; 13 dorsolateral pointed tubercles form a complete longitudinal line on the body; most posterior (13th) pointed dorsolateral tubercle being distinctly largest, slightly posterior to insertion point of hindlimb, projecting backwards; pointed dorsolateral tubercles almost equally spaced, 8th to 12th slightly smaller than others, pointing out almost perpendicularly from body; slightly enlarged, rounded tubercles form weakly elevated curved transversal crests on either side of the vertebral line between 1st and 12th dorsolateral pointed tubercles; slightly enlarged tubercles on dorsal surface of tail, forming two weakly recognizable longitudinal parallel lines; barely defined dorsal pelvic shield in sacral area; lateral surface of body with densely scattered enlarged conical tubercles; venter without enlarged tubercles; scattered, conical tubercles on limbs; no pointed tubercles around cloaca; longitudinal row of distinctly enlarged tubercles lateroventrally on tail, forming a longitudinal row from tail base to three fourth of tail length; no enlarged tubercles on ventral surfaces of tail.

After almost four years in ethanol, flanks, dorsal surfaces of limbs and tail uniformly dark grey-brown; neck with distinct light grey spot; vertebral region mottled with brown and grey; throat grey, venter and ventral surfaces of tail grey-brown. Hemipenes whitish.

*Variation*.— For morphological measurements and proportions see Table 2 and Supporting Information S1. In life, dorsal colouration of head, dorsum and tail light grey. Lateral parts of the body beige, brown or dark brown, with few dark brown spots; limbs almost uniformly dark brown. This pattern is likely to refer to a stress colouration (Fig. 9). When unstressed, most parts of the body beige, with a slightly lighter area on the head anterior to the eyes and in the vertebral region of the dorsum. In preservative, ZSM 2172/2007 is greyish with brown blotches and flecks on dorsum and flanks whereas the other paratypes are more or less uniformly dark brown. The tail base of the males is only slightly more thickened than in the females.

*Genital morphology*.— Everted hemipenes were available from the holotype (ZSM 2170/2007) and from four paratypes (ZSM 2171/2007, 2176–2177/2007, 2179/2007). Structures were characteristic and concordant among all five specimens and are described based on the holotype. Hemipenis length is 3.2 mm. Hemipenis width in the fully turgid state is about 2.3 mm. The retractor muscle is visible through the transparent hemipenis integument and bifurcates after about 1/2 of the hemipenis length. From the wide apex, two narrow tubular processes (0.5 mm in width) extend about 1.1 mm distally, with each end of the retractor muscle ending at the tip of these processes. The end of each of the tubes bears a distinct spine. The apex processes with their large spines are easily recognizable also in incompletely everted hemipenes (Fig. 6). When the hemipenis is everted but not turgid (in preserved specimens), the processes are very distinct and project much more distinctly from the apex.

*Etymology*.— The species epithet is an adjective derived from the Latin “desperatus” meaning “desperate”. Although the known range of the species is within a nature reserve established decades ago, its habitat is in truth barely protected and subject to numerous human-induced environmental problems resulting in severe habitat destruction [41], thus threatening the survival of the species.

*Distribution*.— Only known from the southern edge of Forêt d'Ambre Special Reserve, northern Madagascar.

*Natural History*.— All individuals were found roosting at night on small branches or leaves about 5–100 cm above the ground in disturbed rainforest. Individuals occurred in apparently high abundance and were also found at the border of forest clearings on banana plants. One female laid two large eggs when kept in a plastic bag. When stressed, individuals can quickly change colour and display a broad pale vertebral stripe contrasting with the darker flanks (Fig. 9A).

***Brookesia*** **sp.** A single specimen (ZSM 1508/2008, FGZC 1932), collected in the Ampombofofo region (12°06′S, 49°20′E, 17 m a.s.l., on 12 March 2006 by S. Megson), northern Madagascar, is apparently a subadult and most similar in external morphology to *Brookesia micra*. Its measurements (in mm) are as follows: TL 21.5, SVL 14.5, TAL 7.0, HW 3.0, HH 2.7, ED 1.7, FORL 4.7. It shares with *B. micra* the presence of an indistinct, small pelvic spine, presence of a supernasal cone, only one enlarged tubercle on lateral side of head, absence of supraocular cone, absence of lateral and dorsal spines on tail, and a relative tail length of 0.48. However, it differs from *B. micra* by the absence of dorsolateral spines on body, and slightly in colouration. The lack of countable dorsolateral spines is shared with most examined specimens of *B. minima* and *B. tuberculata*. However, *B. minima* lacks a conspicuous supranasal cone and *B. tuberculata* exhibits a distinct supraocular cone, lacking in this specimen from Ampombofofo. Unfortunately, no molecular data of this specimen were available, but given the combination of morphological characters in other species of the *B. minima* group and their validation by molecular data, we suspect that the population from Ampombofofo may represent another distinct undescribed species of *Brookesia*. However, we refrain from a formal description until more material and molecular data become available.

### Comparative data on genital morphology

In addition to the descriptions and notions of hemipenial morphology provided for the four new species decribed above, we here provide new morphological data on the hemipenes of four described species of the *B. minima* group (see Fig. 6). The genital morphology has been described before for three of these species (*B. peyrierasi*, *B. ramanantsoai*, *B. tuberculata*: [5], [18], [36]) while it was previously unknown for *B. minima*.

#### *B. ramanantsoai*

The hemipenes of one examined specimen (ZSM 10/2009) agree with previous descriptions and are globular structures 3.1 mm in diameter. There are no visible external hemipenis ornamentations. The hemipenes are not fully turgid. Their presumed regular balloon-like shape can therefore only be hypothesized, and the diameter might be an underestimate.

#### *B. minima*

Everted hemipenes have previously not been described for this species, and were newly available for two specimens from Manongarivo, ZSM 1709/2004 and ZSM 990/2003. In both specimens the hemipenis is a large globular structure 3.9 mm in diameter (in ZSM 990/2003), similar to what has been described before for *B. ramanantsoai*. The hemipenis has no visible ornaments and no additional statements regarding its shape can be made because of possible influences of differences in turgidity.

#### *B. peyrierasi*

Various new specimens with everted hemipenes are available from the type locality Nosy Mangabe, and they all agree fully with previous hemipenis descriptions (e.g., [5]). Hemipenes are wide and in the turgid state become wider towards the apex where they are clearly bilobed, with four small spines on each of the lobes. In ZSM 437/2005, hemipenis length is 4.2 mm and maximum hemipenis width is 2.0 mm.

#### *B. tuberculata*

Various new specimens from the type locality Montagne d'Ambre with partly or fully everted hemipenes are available. The hemipenes are especially well displayed in ZSM 1708/2004. They are long tubular structures without ornamentation on pedicel and truncus, with a distinct bulge in the truncus: The hemipenis starts narrow, bulges at about 1/2 of hemipenial length, becoming narrower again, and then again more wide at the apex which bears a typical crown-like structure.

## Discussion

### Integrative taxonomy and species delimitation

**Figure 10:**
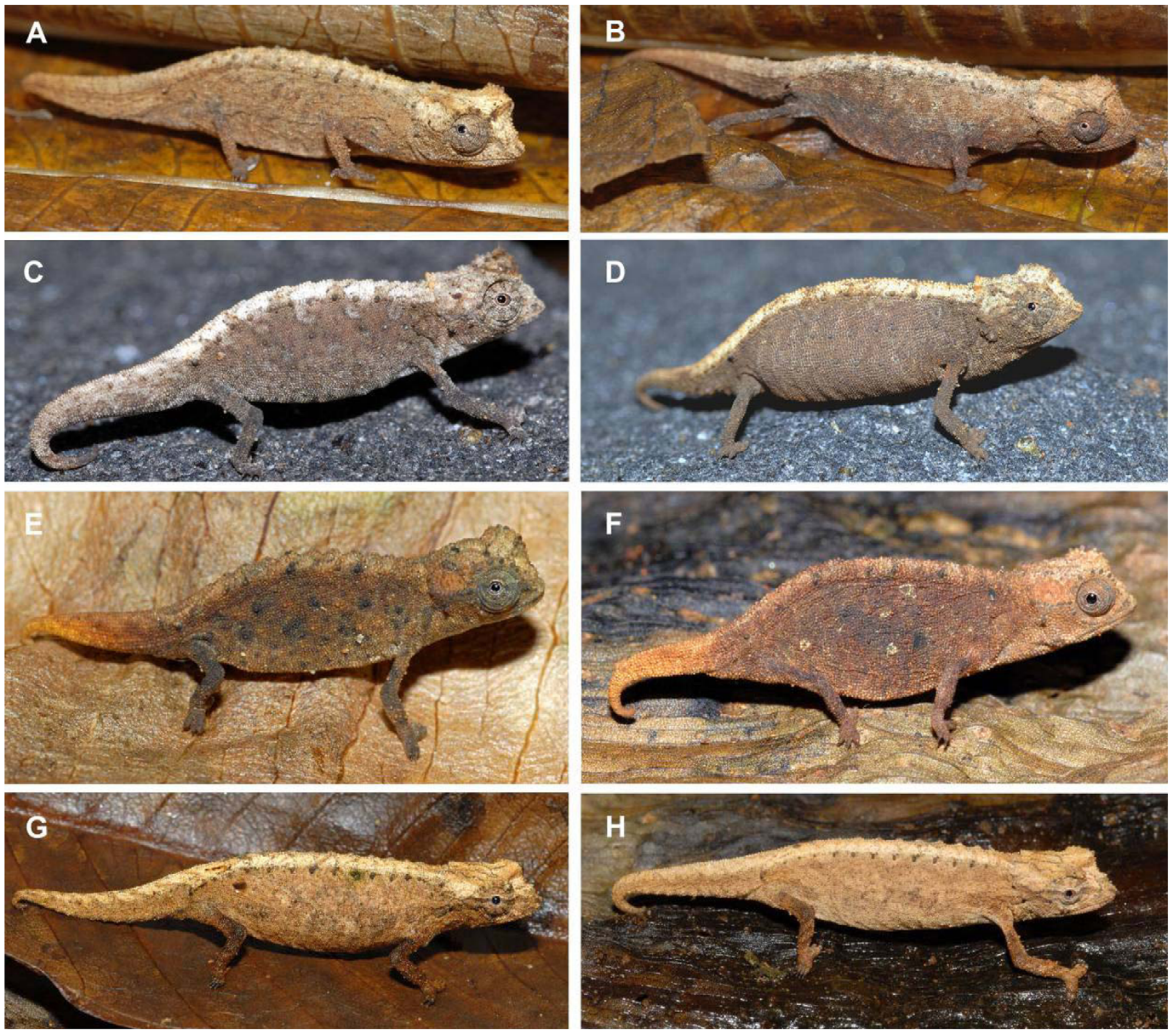
Adult specimens of newly described species in life. (A) male and (B) female of *Brookesia tristis* from Montagne des Français; (C) male and (D) female of *Brookesia confidens* from Ankarana; (E) male and (F) female of *Brookesia micra* from Nosy Hara; (G) male and (H) female *Brookesia desperata* from Forêt d'Ambre.

Taking into account the four new species described herein (Fig. 10), the species diversity of the *Brookesia minima* group has doubled from five species (not including *B. karchei*) accepted in 1996–1999 [5], [20] to currently 11 nominal species and 1–3 additional candidate species: *B. confidens*, *B. dentata*, *B. desperata*, *B. exarmata*, *B. karchei*, *B. micra*, *B. minima*, *B. peyrierasi*, *B. ramanantsoai*, *B. tristis*, *B. tuberculata*, *B.* sp. from Betampona, *B.* sp. aff. *karchei* from Daraina, and *B.* sp. from Ampombofofo. This surprising rise is not explainable by taxonomic inflation, considering that each of these species is well characterized as an independent evolutionary lineage by the concordant signal of nuclear and mitochondrial genes and by morphological characters (Fig. 2 and Fig. 3, Tables 3 and 4). Those species pairs that superficially are most similar in external morphology, such as *B. desperata* and *B. karchei*, *B. confidens* and *B. tristis*, and *B. dentata* and *B. ramanantsoai*, are not sister species in the molecular phylogenies (Fig. 2 and Fig. 3), further supporting their validity as separate evolutionary lineages and species.

In the years since the original definition of the *Brookesia minima* group [18], this group has been usually considered to be monophyletic, although one additional species of comparatively larger size, *B. karchei*, has been demonstrated to be part of this clade [14]. Also, the molecular data have confirmed that *B. dentata* and *B. ramanantsoai* belong to this clade, although they are less miniaturized than most other species in the group. DNA sequences are now available ([14], [17] and the present study) for all described species of Malagasy leaf chameleons except for *B. bekolosy*, a rather large-sized species from Manongarivo which bears no similarity to species of the *B. minima* group. It therefore can be assumed that no other described species of *Brookesia* will in the future be allocated to the *B. minima* group, although the discovery of further undescribed species would not be surprising.

On the contrary, in several other species and species complexes of *Brookesia* more convoluted taxonomic situations are found. The widespread eastern *B. superciliaris* shows strong mitochondrial divergences among populations; however, this structuring is not mirrored by divergences in one nuclear gene studied nor in morphology, suggesting these populations are conspecific [42]. The equally widespread *B. thieli* as well contains several deep genealogical lineages which however do not form a monophyletic group, with the morphologically divergent *B. lineata* and *B. vadoni* clustering within the *B. thieli* clade [14]. Finally, the two morphologically distinct species *B. ambreensis* and *B. antakarana*, both microendemic in Montagne d'Ambre, do not show any consistent mitochondrial or nuclear DNA differentiation [14].

Amongst leaf chameleons, the miniaturized species of the *B. minima* group are those with the most controversial taxonomic history. However, somewhat paradoxically, our integrative taxonomic analysis demonstrates that species delimitation and diagnosis is more straightforward in the *B. minima* group than in many other leaf chameleons.

### Phylogeny, biogeography and morphological divergence between sister species

The concordant and highly supported mitochondrial and nuclear gene trees support the notion that they represent the correct species trees for the *Brookesia minima* group. The results are in strong agreement with geographic distribution but indicate homoplasy of morphological traits. The three main groups consistently recovered, named A, B and C in Figs. 2 and 3, correspond to species either confined to the extreme north of Madagascar, (clade A: *B. confidens*, *B. desperata*, *B. micra*, *B. tristis*, *B. tuberculata*), distributed in the east and north-east (clade B: *B. karchei*, *B. peyrierasi*), or in the west, north-west and northern central east (clade C: *B. dentata*, *B. exarmata*, *B. ramanantsoai*, and an undescribed candidate species from Betampona). The nuclear genes furthermore support a position of *B. minima* at the base of clade A, which again agrees with its distribution pattern given that this species occurs in the Sambirano region, slightly more southwards than the other members of this clade.

The distribution of species in clade C is surprising at first glance, given that species of this group occur both in eastern rainforests of low- and mid-elevation, and in transitional and dry forests of the west. However, in general terms, niche conservatism seems to have played only a minor role in the diversification of the *B. minima* group (see below). The distribution pattern of clade C on the contrary is consistent with the existence of putative “retreat-dispersion watersheds” connecting the north-west and and northern central east of Madagascar. Such watersheds [43] have been proposed to explain distributional patterns mainly of lemurs, and in fact there are examples from other organisms with a distribution area rather similar to that of clade C of the *B. minima* group. However, these examples all involve rather recent dispersal events (e.g., the lemur *Eulemur fulvus*, or the gecko *Phelsuma lineata* [44]–[46]). The distribution of clade C suggests that opportunities for dispersal across Madagascar's northern-central high plateau have existed (either continuously or intermittently) for many millions of years via forests associated with rivers flowing both eastwards and westwards from the plateau. The frog sister species pair *Boophis luteus* (east) and *B. tampoka* (west and northwest) may provide another example of ancient dispersal across this corridor, with subsequent differentiation of *B. tampoka* in its distribution area [47], [48].

Our well supported molecular phylogeny also provides some insights into the evolution of morphological traits within this group. *Brookesia desperata* is deeply nested within clade A and differs in various characters from all other species in this clade: it is larger (25–30 mm vs. 15–24 mm SVL), typically has 13 distinct dorsolateral spines on the body (vs. typically 11 or indistinct), distinct lateral spines on tail (vs. absent), and 3 lateral head tubercles (vs. 1–2, see Table 4). Parsimony-optimization of morphological transformations on the molecular tree (not shown) suggests that the ancestor of clade A did not share these traits. Based on its phylogenetic position in our analysis (highly supported by all genes, and by the combined analysis [Fig. 2 and 3]) there is no doubt that this species indeed belongs to the *B. minima* group, and is not more closely related to other, larger-sized *Brookesia* species. This suggests that the ancestor of *B. desperata* re-evolved a larger body size, and the origin of its other distinctive character states in externally visible structures may be correlated with this size increase. Such a reversal to a larger body size has also been observed in a miniaturized clade of microhylid frogs of the genus *Stumpffia* in the same region of northern Madagascar [49], and in other African frogs [50]. In contrast, clade C consists mainly of rather large-sized species (25–30 mm SVL; Table 3), and the somewhat smaller size of *B. exarmata* (max. 27 mm SVL) appears to be a derived trait within this clade.

*Brookesia micra* is morphologically distinct from all other species in the *B. minima* group in several respects. Relative to SVL, it has the shortest tail and the largest head (Table 3; Fig. 4). At the same time, this is the smallest species of the group, and because short tails and large heads are typically found in juvenile lizards, it is tempting to interpret these traits in *B. micra* as paedomorphic features. However, a clear trend of increasing head size and decreasing tail length with decreasing body length is not evident in the *B. minima* group (Fig. 4). For instance, *B. minima* is only 2 mm larger than *B. micra* in average male SVL but has the smallest relative head size in the whole group. And the small-sized *B. peyrierasi* and *B. tuberculata* have distinctly longer tails than the much larger *B. desperata*. Phylogenetically, character states for these traits (tubercles and spines, head size or tail length) do not constitute convincing morphological synapomorphies for any clade or subclade in the *B. minima* group.

Genital morphology is useful for species delimitation, and all sister species in the *B. minima* group for which hemipenial data were available (Fig. 6) are characterized by clear genital differences. The clade composed of *B. desperata* and *B. tristis* might be characterized by the presence of terminal spines on the hemipenes, and the clade of *B. tuberculata* and *B. confidens* has tubular narrow hemipenes. On the other hand, both *B. minima* and *B. ramanantsoai* have hemipenes that in the fully everted state are globular and free of any ornaments, yet these two species are not closely related. This suggests that hemipenial characters might not be particularly useful to reconstruct species-level phylogenies in *Brookesia*, although they have provided valuable synapomorphies for other major groups within the Chamaeleonidae [51].

Phylogenetic niche conservatism predicts that closely related species will be similar in their ecological niches, and has recently gained attention as a possible factor influencing clade diversification (e.g., [52], [53]). However, within numerous clades of organisms obvious niche conservatism is found at only some, but not all, phylogenetic levels, and this complicates the assessment and analysis of this factor [54]. A detailed analysis of niche conservatism within the *B. minima* group is beyond the scope of the present study, but a few general observations are possible. First, all species of the *B. minima* group appear to be strict specialists of particular habitats, occurring either in rainforest or dry forest, and at a very limited elevational range: Those species known from more than one locality occur either in lowland (*B. peyrierasi*, *B. minima*) or mid-elevational rainforest (*B. ramanantsoai*). *Brookesia tuberculata*, from the elevationally diverse Montagne d'Ambre Massif, has only been recorded at 900–1000 m elevation [5], [55]. Furthermore, all species in the *B. minima* group appear to be allopatrically distributed, although parapatric or even sympatric occurrence cannot be excluded for *B. desperata* and *B. tuberculata*. These two species occur at different elevations in a continuous forest block of the Montagne d'Ambre Massif, and their localities are only a few kilometres apart. Second, despite the apparently narrow ecological tolerance of individual *B. minima* group species, the group as a whole has colonized a wide variety of habitats in Madagascar, from rainforest to dry forest and from near sea level to at least 1200 m (*B. ramanantsoai* at Mandraka). The phylogenetic tree indicates recurrent shifts of habitat specialization: for instance, in clade A *B. tuberculata* from mid-altitude rainforest habitat has as close relatives species specialized to drier low-elevation habitat. Similarly, within clade C the mid-altitude rainforest species *B. ramanantsoai* is placed close to the low-elevation dry forest specialist *B. exarmata*.

### Microendemism in the *Brookesia minima* group

Species of the *B. minima* group are known from uniformly very small distribution areas. Several species are supposed to occur at multiple localities, e.g., *B. peyrierasi* from the Masoala Peninsula and the small nearby offshore island Nosy Mangabe; *B. minima* from its type locality on the offshore island Nosy Be and the Manongarivo massif on the adjacent mainland; *B. ramanantsoai* from Mandraka and Andasibe, and *B. dentata* from its type locality Maevatanana and from the nearby Ankarafantsika National Park. However, molecular data in these species are so far available from one locality each, and in the cases of *B. dentata*, *B. minima*, and *B. ramanantsoai*, the available DNA sequences are not from the topotypical specimens. Given the high degree of genetic divergence among morphologically similar species of the *B. minima* group, it is quite possible that species known from more than one locality might comprise further undescribed candidate species. The only species for which molecular data are available from more than one site is *B. karchei* where specimens from Daraina are genetically strongly divergent from those collected at the type locality Marojejy, and are therefore here named *B.* sp. aff. *karchei*, pending further study (Fig. 3; sequences available only for *CMOS*).

Microendemism is extreme in the four new species described herein, each of which is known only from its respective type locality. For each of them our analysis includes mitochondrial DNA sequences for a large series of specimens (Fig. 1). Compared to the very deep divergences between species, the *ND2* within-population differentiation in all four species is relatively low (<3% vs. >17%), and we detected no sharing of deeply divergent haplotypes within a population. The high molecular divergences among species thus appear best explained by low vagility and genetic isolation of local populations in these miniaturized species, rather than an exceptionally fast molecular substitution rate. Only few studies have dealt with individual dispersal and home ranges of chameleons, although movements over relatively long distances have been observed in males of larger chameleon species [56], [57], e.g. more than 100 m in 12 days in the Malagasy *Furcifer pardalis* [57]. No studies have so far tested the possibility of a very low individual dispersal capacity of *Brookesia*.

In summary, various lines of evidence identify the *B. minima* group as an ancient clade of deeply differentiated and well delimited species with extremely small geographic distributions, whose diversification was driven by low vagility, and thus lack of gene flow between small and isolated populations. This is suggested by (1) their deep molecular differentiation, with no haplotype sharing among species or populations in nuclear or mitochondrial genes, and concordant phylogenetic signals from independent nuclear and mitochondrial genes, (2) the pattern of regional diversification, with subclades restricted to particular regions of Madagascar, and (3) the presence of consistent differences in external and genital morphology among all species. According to a molecular dating analysis [14], the diversification of this group took place in the Eocene to Oligocene, with sister species diverging >10 million years ago (*B. desperata/B. tristis*) to >20 mya (all other pairs of sister species; Fig. 3). We hypothesize that these species have been microendemic to the same small distribution ranges for this extended period of time, with a low prevalence of range shifts, and probably with repeated local extinctions of populations.

It has been noted that most of the 15 *Brookesia* species in northern Madagascar show a remarkable degree of microendemism [12]. The importance of northern Madagascar has also been assessed based on an explicit analysis of species richness of *Brookesia*, which was highest in this part of the island [14]. Considering the novel classification and new species of the *B. minima* group, this trend is now even more obvious, with 21 species of *Brookesia* being confined to northern Madagascar [12].

Microendemism is widespread in Malagasy animals, but there are few examples in which it is as extreme as in the miniaturized species of the *Brookesia minima* group and in frogs of the genus *Stumpffia* [49]. This indicates a possible body size effect on the degree of microendemism, which is consistent with the body-range size relationship previously demonstrated in the Malagasy mantellid frog radiation [58]. In general, a correlated decrease in species' ranges with decreasing body size is a recognized pattern in animals [59]. A closer analysis of this pattern in Madagascar's biota may yield new insights and provide at least a partial explanation for the prevalence of microendemic patterns in some groups of animals and the absence of such patterns in others.

### Ecological and life-history correlates of miniaturization

A previous study found *Brookesia* from northern Madagascar to be confined almost entirely to rainforest, with only one species, *Brookesia stumpffi*, also found in the dry forests of the limestone canyons at Ankarana [12]. *Brookesia ebenaui*, hitherto considered a rainforest species, was later also discovered in karstic dry forest at Montagne des Français [41]. In addition to these larger leaf chameleon species, three miniaturized species from northern Madagascar (*B. confidens*, *B. micra*, and *B. tristis*) plus one species from western Madagascar (*B. exarmata*) are specialized to dry forest on karstic underground, indicating that this habitat type is much more important than hitherto assumed for these tiny reptiles. One miniaturized species, *B. dentata*, occurs in dry forest on non-karstic soil.

In general, in Madagascar, miniaturization of lizards does not appear to be restricted to tropical humid environments. Dwarf geckos of the genus *Lygodactylus* occur in montane environments above 2300 m elevation and are very common in dry western forests where they can reproduce all year round [60]. Geckos lay eggs with hard calcareous shells that are less prone to desiccation than the soft shells of chameleon eggs. Smaller body size is correlated with a proportionally higher body surface area and thus higher risk of desiccation, probably even when scales cover the skin, as in squamate reptiles. Given that the climate of Madagascar is characterized by unpredictability at various scales [61], the presence of miniaturized lizards in dry environments is surprising. Probably the rather accentuated rainy season even in the west of Madagascar, together with typically low temperatures in the dry season and the humidity buffer provided by leaf litter and fissures in karstic soil provide a microclimate that has allowed these species to survive under superficially adverse conditions.

Extremely small animals often display unique behavioural repertoires, and evolutionary novelty may also arise through ecological specialization directly related to size. For instance, miniaturized frog species specialize in very small prey that often largely consists of mites and ants. Several species from these arthropod groups contain alkaloids, and their consumption may have constituted an important prerequisite for the evolution of skin alkaloid sequestering, aposematic colour and diurnal behaviour in poison frogs [62]. Miniaturized frog species also tend to display derived life history traits [3] with a tendency toward fewer and larger offspring, often correlated with the loss of a free-living, feeding tadpole stage [63]. In snakes and possibly other amniotes, allometry of the body and of organ sizes probably limits the proportion of the body cavity available for the formation of eggs, so that the smallest snakes typically have a rather stout and short body. Only one egg or young is typically produced, with this single offspring reaching a relatively large 50% of adult size vs. 10% in larger related species [2]. Minimum sizes of adult amniotes may therefore be defined by a tradeoff between number and size of offspring [2]. In this study we report a clutch size of two eggs in both *B. desperata* and *B. tristis*, as it has also been found for *B. exarmata* [20]. Several species of larger-sized *Brookesia*, including *B. antakarana*, *B. brygooi*, *B. decaryi*, *B. griveaudi*, and *B. stumpffi*, have clutch sizes of 2–5 eggs, with average clutch size in *B. antakarana* being 4 eggs [64]. These findings are consistent with a hypothesis of reduced clutch sizes in miniaturized tetrapods.

Egg length in miniaturized *Brookesia* is 5.8–5.9 mm in *B. tristis* (this study), 8 mm in *B. exarmata* [20], and 2.5 mm in *B. minima* [64]. Egg length in larger *Brookesia* is considerably longer (17 mm in *B. griveaudi* and 14 mm in *B. stumpffi*), but the relative size of the eggs seems to be similar among non-miniaturized and miniaturized species: the ratio of egg length/female TL is ca. 0.16–0.17 in *B. griveaudi* and *B. stumpffi* (both larger species outside the *B. minima* group), and in the *B. minima* group ranges from 0.08 (*B. minima*) to 0.17 (*B. tristis*) and 0.20 (*B. exarmata*). However, these comparisons should be interpreted very cautiously, as the size of chameleon eggs often significantly increases after their deposition through intake of humidity, and the reported (often anecdotal) values might therefore not be directly comparable.

### Island effects and records of extreme miniaturization

The geckos *Sphaerodactylus ariasae* and *S. parthenopion* (maximum SVL in both sexes 18 mm) are considered to be the worldwide smallest of the approximately 8,000 squamate and 23,000 amniote species [1]. Several species of the *B. minima* group approach this size (Table 3), and with a maximum male SVL of 16 mm and a maximum total length (TL) below 30 mm (in both sexes), *Brookesia micra* is certainly one of the smallest amniotes on the globe (Fig. 8).

Whether the extreme miniaturization of *Brookesia micra* can be interpreted as a case of island dwarfism is uncertain. Madagascar as a very large island is well known for several cases of gigantism (e. g. extinct elephant birds of the genus *Aepyornis*, giant lemurs and giant tortoises) but harbours even more remarkable lineages of dwarfs. The nocturnal mouse lemurs (genus *Microcebus*) include a few species (e. g. *M. berthae*) which are among the smallest primates in the world [44]. Among the amphibians, cophyline microhylids of the genus *Stumpffia* represent a large radiation with about 40 identified species and candidate species [34], [49], [65], including some of the world's smallest frogs (e. g., *S. tridactyla, S. pygmaea*). An independent extreme miniaturization event occurred in a still undescribed cophyline genus from southeastern Madagascar [65]. Among chameleons, the species of the *B. minima* group are by far the smallest species, and the extremely small size of *B. micra* could represent a “double” island dwarf effect. In this scenario, Madagascar as a large island led to the evolution of the *Brookesia minima* group whereas the isolated karstic block now representing the islet Nosy Hara, might have favored the extreme miniaturization found in *B. micra*. However, since the sea between Nosy Hara and the adjacent mainland of Madagascar is shallow (depth <25 m) this island must have been connected with Madagascar during the last glaciation. Future surveys are needed to clarify whether this species is really endemic to Nosy Hara or more widely distributed. Interestingly, such a “double island effect” could also be applicable to *Sphaerodactylus ariasae* which is known from the small islet Isla Beata and adjacent areas of Hispaniola [1].

Considering the available data, which species are actually the smallest vertebrates, amniotes and tetrapods on Earth? If all tetrapods are considered, the record clearly belongs to the anurans. However, identifying which is the smallest species is futile and strongly depending on the measuring scheme. All species competing for the title of smallest frog (and thus smallest tetrapod) in the world (*Eleutherodactylus iberia*, *Brachycephalus didactylus*, *Stumpffia pygmaea*, *S. tridactyla*, and others) possess SVL values around 8–10 mm [66], whereas the smallest salamanders of the genus *Thorius* mature at 16–17 mm SVL and ca. 36–37 mm TL [67].

Among amniotes, the smallest species are lizards, but they are consistently larger than amphibians, suggesting that different constraints are acting on this group. The maximum recorded SVL of *Sphaerodactylus ariasae* (18 mm) is below the maximum value in *Brookesia micra* (19.9 mm). However if using TL as yardstick, the record of the smallest squamate and amniote in fact applies to *B. micra* with a maximum TL of 29 mm, given that *S. ariasae* has a TL well above 30 mm [1] due to its relatively longer tail. The ‘award’ for the lower size record in amniotes, as in amphibians, therefore depends on the type of measurement used for ranking.

## Supporting Information

Supporting Information S1 **Iincluding morphological determination key, Table S1 (voucher specimens and Genbank accession numbers), Table S2 (divergence-date calibration priors), Table S3 (additional morphometric measurements), Table S4 (additional morphological data), Figure S1 (chronogram), Fig. S2 (** ***B. minima*** **genital morphology), Fig. S3 (holotype of** ***B. tristis*** **), Fig. S4 (holotype of** ***B. confidens*** **), Fig. S5 (holotype of** ***B. micra*** **), Fig. S6 (holotype of** ***B. desperata*** **).**
(PDF)
