# Supplementary material for: Rivaling the World's Smallest Reptiles: Discovery of Miniaturized and Microendemic New Species of Leaf Chameleons (Brookesia) from Northern Madagascar
Source: PLoS One. 2012 Feb 14;7(2):e31314. doi: 10.1371/journal.pone.0031314 (PMC3279364; doi:10.1371/journal.pone.0031314)
Supplement: Supporting Information S1 — Iincluding morphological determination key, Table S1 (voucher specimens and Genbank accession numbers), Table S2 (divergence-date calibration priors), Table S3 (additional morphometric measurements), Table S4 (additional morphological data), Figure S1 (chronogram), Fig. S2 ( B. minima genital morphology), Fig. S3 (holotype of B. tristis ), Fig. S4 (holotype of B. confidens ), Fig. S5 (holotype of B. micra ), Fig. S6 (holotype of B. desperata ). (PDF) [file pone.0031314.s001.pdf]

Supporting information of the manuscript:

Rivaling the World's Smallest Reptiles: Discovery of Miniaturized and Microendemic New Species of Leaf Chameleons (*Brookesia*) from Northern Madagascar

Frank Glaw, Jörn Köhler, Ted M. Townsend, Miguel Vences

Content:

Table S1. List of voucher specimens and GenBank accession numbers for all DNA sequences included in the analysis.

Table S2. Divergence-date calibration priors.

Table S3. Morphometric measurements (in mm) of additional examined specimens of species in the *Brookesia minima* group.

Table S4. Variation of selected morphological characters in examined specimens of the *Brookesia minima* group.

Morphological identification key to the species of the *Brookesia minima* group

Supplementary figures S1-S6

Table S1. List of voucher specimens and GenBank accession numbers for all DNA sequences included in the analysis.

Species	Voucher #	ND2	16S
**Outgroups**			
Brookesia brygooi	FGZC 689 (ZSM 12/2006)	FJ975191	KUZ R61408 JN674044
Brookesia nasus	FGMV 445 (ZSM 728/2003)	FJ975158	HQ13509
Brookesia superciliaris	HDZ-CCR BM 24	AF448778	UADBA 20730 HQ130513
**Miniaturized clade**			
Brookesia confidens	FGZC 1194	JN673976	--
Brookesia confidens	FGZC 1195	JN673981	--
Brookesia confidens	FGZC 1196	JN673978	JN674045
Brookesia confidens	FGZC 1197	JN673977	--
Brookesia confidens	FGZC 1198	JN673979	--
Brookesia confidens	FGZC 1199	JN673980	--
Brookesia dentata	KUZ R58383	FJ975174	JN674046
Brookesia desperata	FGZC 1250	JN673996	--
Brookesia desperata	FGZC 1251	JN673997	--
Brookesia desperata	FGZC 1252	JN673998	--
Brookesia desperata	FGZC 1253	JN673999	--
Brookesia desperata	FGZC 1255	JN673982	--
Brookesia desperata	FGZC 1256	JN673983	--
Brookesia desperata	FGZC 1257	JN673984	--
Brookesia desperata	FGZC 1258	JN673985	--
Brookesia desperata	FGZC 1259	JN673986	--
Brookesia desperata	FGZC 1260	JN673987	--
Brookesia desperata	FGZC 1261	JN673988	--
Brookesia desperata	FGZC 1262	JN673989	--
Brookesia desperata	FGZC 1263	JN673990	--
Brookesia desperata	FGZC 1264	JN673991	--
Brookesia desperata	FGZC 1265	JN673992	--
Brookesia desperata	FGZC 1266	JN673993	--
Brookesia desperata	FGZC 1267	JN674003	--
Brookesia desperata	FGZC 1268	JN673994	--
Brookesia desperata	FGZC 1269	JN673995	--
Brookesia desperata	FGZC 1700	JN674000	--
Brookesia desperata	FGZC 1879	JN674001	--
Brookesia desperata	FGZC 1880	JN674002	JN674047
Brookesia exarmata	FGZC 744	JN674004	--
Brookesia exarmata	FGZC 816 (ZSM 72/2006)	FJ975175	JN674048
Brookesia exarmata	FGZC 965 (ZSM 147/2006)	FJ975176	--
Brookesia karchei	UADBA 27293	FJ975169	--
Brookesia karchei	FGZC 2005.2752 (ZSM 125/2005)	FJ975170	JN674049
Brookesia micra	FGZC 1270	JN674005	--
Brookesia micra	FGZC 1271	JN674006	--
Brookesia micra	FGZC 1272	JN674007	--
Brookesia micra	FGZC 1273	JN674008	--

Brookesia micra	FGZC 1274	JN674009	--
Brookesia micra	FGZC 1275	JN674010	--
Brookesia micra	FGZC 1276	JN674011	--
Brookesia micra	FGZC 1277	JN674012	--
Brookesia micra	FGZC 1278	JN674013	--
Brookesia micra	FGZC 1279	JN674014	--
Brookesia micra	FGZC 1280	JN674015	--
Brookesia micra	FGZC 1281	JN674016	--
Brookesia micra	FGZC 1282	JN674017	--
Brookesia micra	FGZC 1830	JN674018	--
Brookesia micra	FGZC 1831	JN674019	JN674050
Brookesia micra	FGZC 1832	JN674020	--
Brookesia micra	FGZC 1833	JN674021	--
Brookesia micra	FGZC 1834	JN674022	--
Brookesia minima	FGMV 724	JN674023	--
Brookesia minima	FGMV 725	JN674024	--
Brookesia minima	UADBA FGMV 786	FJ975164	JN674051
Brookesia minima	UADBA FGMV 787	FJ975165	--
Brookesia peyrierasi	ZCMV 2189	JN674025	--
Brookesia peyrierasi	ZCMV 2190	JN674026	--
Brookesia peyrierasi	ZCMV 2191 (ZSM435/2005)	FJ975171	--
Brookesia peyrierasi	ZCMV 2192 (ZSM436/2005)	FJ975172	JN674052
Brookesia peyrierasi	ZCMV 2193	JN674027	--
Brookesia peyrierasi	ZCMV 2194	JN674028	--
Brookesia peyrierasi	ZCMV 2195	JN674029	--
Brookesia ramanantsoai	ZSM 10/2009	FJ975173	JN674053
Brookesia sp. “Betampona”	HDZ-CCR BET 11	FJ975162	--
Brookesia sp. “Betampona”	HDZ-CCR BET 130	FJ975163	JN674054
Brookesia tristis	UADBA FGZC 2004.477	FJ975167	--
Brookesia tristis	UADBA FGZC 2004.478	FJ975168	JN674055
Brookesia tristis	FGZC 1101	JN674030	--
Brookesia tristis	FGZC 1187	JN674031	--
Brookesia tristis	FGZC 1188	JN674032	--
Brookesia tristis	FGZC 1189	JN674033	--
Brookesia tristis	FGZC 1190	JN674034	--
Brookesia tristis	FGZC 1191	JN674035	--
Brookesia tristis	FGZC 1192	JN674036	--
Brookesia tristis	FGZC 1656	JN674037	--
Brookesia tristis	FGZC 1657	JN674038	--
Brookesia tristis	FGZC 1734	JN674039	--
Brookesia tuberculata	UADBA FGZC 2004.479	FJ975166	JN674056
Brookesia tuberculata	FGZC 1081	JN674040	--
Brookesia tuberculata	FGZC 1082	JN674041	--
Brookesia tuberculata	FGZC 1083	JN674042	--
Brookesia tuberculata	FGZC 1119	JN674043	--

Species	Voucher #	CMOS	RAG1
**Outgroups**			
Brookesia brygooi	KUZ R61408	FJ984305	FJ984235
Brookesia nasus	FGMV 445 (ZSM 728/2003)	FJ984273	FJ984202
Brookesia superciliaris	UADBA 20730	FJ984303	FJ984233
**Miniaturized clade**			
Brookesia confidens	FGZC 1196	JN674057	JN674060
Brookesia dentata	KUZ R58383	FJ984289	FJ984218
Brookesia desperata	FGZC 1880	JN674058	JN674061
Brookesia exarmata	FGZC 816 (ZSM 72/2006)	FJ984291	FJ984219
Brookesia karchei	FGZC 2005.2752 (ZSM 125/2005)	FJ984285	FJ984214
Brookesia micra	FGZC 1831	JN674059	JN674062
Brookesia minima	UADBA FGMV 786	FJ984280	FJ984208
Brookesia peyrierasi	ZSM 435/2005 (ZCMV 2005.2191)	FJ984286	FJ984215
Brookesia ramanantsoai	ZSM 10/2009	FJ984288	FJ984217
Brookesia sp. "Betampona"	HDZ-CCR BET 11	FJ984277	FJ984207
Brookesia tristis	UADBA FGZC 2004.477	FJ984282	FJ984211
Brookesia tuberculata	UADBA FGZC 2004.479	FJ984281	FJ984210

Table S2. Divergence-date calibration priors.
The second column gives the actual zero-offset and parameter values for the translated lognormal (TL) distribution as entered in BEAUti [S1]. The third column describes the effective median and upper and lower bounds of the 95% confidence interval (CI), given in millions of years ago (Mya), produced by each TL distribution. Unless otherwise stated, all stratigraphic dates used for calibration purposes are from Gradstein et al. (2004) [S2].

Node	TL zero-offset (mean, st.dev.)	Median (95% CI) (Mya)
1	224 (2.0, 1.2)	231 (225, 301)
2	161 (1.8, 1.2)	167 (162, 204)
3	110 (1.8, 1.3)	116 (111, 187.3)
4	110 (1.8, 1.3)	116 (111, 187.3)
5	70 (1.8, 1.0)	76 (71, 113)
6	70 (1.8, 1.0)	76 (71, 113)
7	70 (1.2, 1.9)	73 (70, 208)
8	69 (1.8, 1.0)	72 (70, 125)

Calibration nodes.

Node 1. The oldest known rhynchocephalians [S3,S4] from the Ladinian-Carnian boundary (228 ± 2.0 Mya) were used to calibrate the node defining Lepidosauria (Rhynchocephalia [represented by *Sphenodon punctatus*] and Squamata [all other sampled taxa]).
Node 2. The stem scincomorph *Balnealacerta* [S5,S6] from the Middle Jurassic (164.7 ± 4.0 Mya) was used to calibrate the node defining Unidentata (all squamates except Gekkota and Dibamidae). We note that Scincomorpha is traditionally defined to encompass Scincidae, Cordylidae, Xantusiidae, Teiidae, Gymnophthalmidae, and Lacertidae, which is a paraphyletic assemblage in our tree. There is some disagreement amongst morphologists/paleontologists as to which subgroup(s) among these taxa *Balnealacerta* and other "scincomorphs" from the same site are more closely related to. From the possible alternatives, we chose the deepest (i.e., more conservative in terms of pushing divergence dates further into the past) node for our calibration point.
Node 3. Stem teiids including *Ptilodon* and other indeterminate taxa [S7,S8] from the Aptian-Albian boundary (112 ± 1.0 Mya) were used to calibrate the node defining Laterata (Teiioidea, Amphisbaenia, and Lacertidae).
Node 4. The stem amphisbaenian *Hodzhhakulia* [S9] from the Aptian-Albian boundary (112 ± 1.0 Mya) was used to calibrate the node defining Lacertibaenia (Amphisbaenia and Lacertidae).
Node 5. The possibly stem varanid genus *Palaeosaniwa* [S10] from the Campanian (70.6 ± 0.6 Mya) was used to calibrate the node defining Paleoanguimorpha (Shinisauridae, Lanthanotidae, and Varanidae).
Node 6. The anguid *Odaxosaurus* [S10,S11] from the Campanian (70.6 ± 0.6 Mya) was used to calibrate the node representing the most recent common ancestor (MRCA) of Xenosauridae and Anguidae.
Node 7. The node defining crown Iguania was calibrated using Campanian (70.6 ± 0.6 Mya) fossils of the stem acrodont iguanian clade Priscagaminae [S12].
Node 8. The crown-group pleurodont iguanian *Saichangurvel* [S13] from the Late Campanian (70.6 ± 0.6 Mya) was used to calibrate the node defining crown Pleurodonta.

Table S2 References

S1. Drummond AJ, Rambaut A (2007) BEAST: Bayesian evolutionary analysis by sampling trees. BMC Evol Biol 7: 214.

S2. Gradstein FM, Ogg JG, Smith AG (2004) A Geologic Time Scale 2004. Cambridge, UK: Cambridge University Press.

S3. Sues HD, Olsen PE (1990) Triassic vertebrates of Gondwanan aspect from the Richmond Basin of Virginia. Science 249: 1020-1023.

S4. Evans SE, Prasad GVR, Manhas BK (2001) Rhynchocephalians (Diapsida : Lepidosauria) from the Jurassic Kota Formation of India. Zool J Linn Soc 133: 309-334.

S5. Evans SE (1993) Jurassic lizard assemblages. In: Buffetaut E, Mazin J-M, editors. Proceedings of the Second Georges Cuvier Symposium, Revue de Paleobiologie, Special Volume 7. pp. 55-65.

S6. Evans SE (1998) Crown-group lizards from the Middle Jurassic of Britain. Palaeontographica A 250: 1-32.

S7. Winkler DA, Murry PA, Jacobs LL (1990) Early Cretaceous (Comanchean) vertebrates of central Texas. J Vert Paleontol 10: 95-116.

S8. Nydam RL, Cifelli RL (2002) Lizards from the Lower Cretaceous (Aptian-Albian) Antlers and Cloverly Formations. J Vert Paleontol 22: 286-298.

S9. Gao KQ, Nessov LA (1998) Early Cretaceous squamates from the Kyzylkum Desert, Uzbekistan. Neues Jahrbüch für Geologische und Paläontologische Abhandlungen 207: 289-309.

S10. Bryant LJ (1989) Non-dinosaurian lower vertebrates across the Cretaceous-Tertiary boundary in northeastern Montana. University of California Publications in Geological Sciences 134: 1-107.

S11. Sullivan RM, Lucas SG (1996) *Palaeoscincosaurus middletoni*, new genus and species (Squamata: Scincidae) from the early Paleocene (Puercan) Denver Formation, Colorado. J Vert Paleontol 16: 666-672.

S12. Gao KQ, Norell MA (2000) Taxonomic composition and systematics of Late Cretaceous lizard assemblages from Ukhaa Tolgod and adjacent localities, Mongolian Gobi Desert. Bull Am Mus Nat Hist 249: 1-118.

S13. Conrad JL, Norell MA (2007) A complete Late Cretaceous iguanian (Squamata, Reptilia) from the Gobi and identification of a new iguanian clade. Am Mus Novit: 1-47.

Table S3. Morphometric measurements (in mm) of additional examined specimens of species in the *Brookesia minima* group.
Note that data for the newly described species are reported in Table 1 of main paper. Used abbreviations: M = male, F = female, J = juvenile; TL = total length; TaL = tail length; HW = head width; HH = head height; Eye = horizontal diameter of eye; rmL = arm length.

Species / voucher number	type status	Sex	TL	SVL	TaL	HW	HH	ED	FORL
Brookesia exarmata									
ZSM 147/2006		F	40.1	25.7	14.4	4.3	4.1	2.3	8.0
ZSM 72/2006		F	39.8	26.5	13.3	4.6	4.3	2.4	8.7
Brookesia minima									
ZSM 443/2000		F	33.3	21.0	12.3	3.2	3.1	2.0	5.9
ZSM 990/2003		M	33.9	20.6	13.3	3.4	2.8	2.2	5.3
ZSM 1709/2004		M	29.9	17.8	12.1	2.8	2.6	1.5	5.4
ZSM 814/2003		F	35.6	21.8	13.8	3.6	3.3	2.0	6.9
Brookesia ramanantsoai									
ZSM 10/2009		M	39.0	21.7	17.3	3.5	3.6	1.8	6.5
ZSM 637/2000		F	43.5	24.9	18.6	3.7	3.7	2.1	7.6
ZSM 638/2000		F	42.9	24.9	18.0	4.0	4.5	2.1	8.3
Brookesia karchei									
ZSM 125/2005		F	51.0	30.7	20.3	5.3	4.9	3.0	10.4
Brookesia peyrierasi									
ZSM 434/2005		F	41.8	27.3	14.5	4.7	4.3	2.5	7.9
ZSM 433/2005		F	43.1	27.4	15.7	4.7	4.7	2.5	7.9
ZSM 438/2005		F	40.6	25.2	15.4	4.4	4.2	2.3	8.3
ZSM 436/2005		M	39.0	22.3	16.7	3.9	3.5	2.4	6.5

ZSM 437/2005	M	37.2	21.3	15.9	3.6	3.2	2.2	6.7
ZSM 4365/2005	M	34.2	19.7	14.5	3.7	3.4	2.1	7.4
Brookesia tuberculata								
ZSM 498/2000	M	30.6	17.0	13.6	3.3	2.7	1.8	5.6
ZSM 499/2000	M	32.1	18.1	14.0	3.4	2.6	1.7	5.1
ZSM 1039/2003	M	33.6	18.8	14.8	3.4	2.6	1.7	5.6
ZSM 1038/2003	M	32.2	18.3	13.9	3.4	2.8	1.9	4.4
ZSM 356/2004	F	28.9	18.2	10.7	3.6	3.0	1.7	4.9
ZSM 1708/2004	M	32.9	18.2	14.7	3.2	2.9	1.9	5.5
ZSM 2098/2007	M	30.7	17.3	13.4	3.5	2.9	1.8	5.8
ZSM 2097/2007	M	31.4	18.7	12.7	3.5	3.0	1.8	5.7
ZSM 2099/2007	M	32.5	18.5	14.0	3.5	3.0	1.8	6.1
ZSM 2100/2007	M	32.8	18.6	14.2	3.3	2.9	2.1	5.7
ZSM 2096/2007	F	34.6	21.7	12.9	4.5	3.9	1.9	6.1
ZSM 2114/2007	J	17.4	10.0	7.4	2.4	2.2	1.4	3.5

Table S4. Variation of selected morphological characters in examined specimens of the *Brookesia minima* group.

species	number of dorsolateral spines on body	pelvic spine	supraocular cone	supranasal cone	lateral spines on tail	dorsal spines on tail	number of enlarged tubercles at lateral side of head	posterior crest
B. minima								
ZSM 990/2003	not countable	indistinct	no	ambiguous	indistinct	no	1	yes
ZSM 1709/2004	14 (indistinct)	indistinct	no	ambiguous	yes	yes	1	yes
ZSM 17/1921	not countable	no	no	ambiguous	no	no	2	indistinct
B. ramanantsoai								
ZSM 638/2000	4 (irregular)	yes	no	yes	no	no	0	indistinct
ZSM 637/2000	4 (irregular)	yes	no	yes	no	no	1	indistinct
ZSM 10/2009	4 (irregular)	yes	yes	yes	no	no	0	indistinct
ZSM 285/2010	8 (left), 11 (right)	yes	yes	yes	no	no	2	no
B. dentata (based on photos of A. Mori)	10-11	yes	no	yes	indistinct	distinct	2-3	yes
B. peyrierasi								
ZSM 447/2010	10-11 (indistinct)	yes	indistinct	yes	indistinct	no	1	yes
ZSM 430/2005	10 (indistinct)	yes	no	yes	no	no	1	indistinct
ZSM 437/2005	10 (indistinct)	yes	no	yes	no	no	1	indistinct
ZSM 433/2005	11	yes	no	yes	no	no	1	indistinct
ZSM 434/2005	10	yes	no	yes	yes	no	1	yes
ZSM 438/2005	10 (indistinct)	yes	no	yes	yes	no	1	indistinct
ZSM 435/2005	not countable	yes	no	yes	indistinct	no	1 (indistinct)	indistinct
B. exarmata								
ZSM 72/2006	12 (small)	yes	no	yes	no	no	1	yes
ZSM 147/2006	11 (indistinct)	yes	no	yes	no	no	1	indistinct
B. tuberculata	indistinct	small	yes	yes	no		1	yes
ZSM 1708/2004	9 (indistinct)	yes	yes	yes	yes	no	2	yes
ZSM 2098/2007	not countable	yes	yes	yes	yes	no	1	indistinct
ZSM 2100/2007	not countable	indistinct	yes	yes	no	no	1	indistinct
ZSM 2097/2007	not countable	indistinct	yes	yes	yes	no	2	indistinct

ZSM 2099/2007	not countable	indistinct	yes	yes	no	no	1	indistinct
ZSM 2096/2007	not countable	yes	yes	yes	indistinct	no	1	indistinct
B. karchei								
ZSM 125/2005	13	yes	yes	yes	yes	indistinct	1	yes
B. desperata								
ZSM 2170/2007 (HT)	13	yes	indistinct	yes	strong	indistinct	3	yes
ZSM 2171/2007	13	yes	indistinct	yes	strong	yes	3	yes
ZSM 2172/2007	13	yes	yes	yes	strong	no	3	yes
ZSM 2173/2007	14	yes	indistinct	yes	strong	yes	3	yes
ZSM 2174/2007	13	yes	indistinct	yes	strong	indistinct	3	yes
ZSM 2175/2007	12	yes	indistinct	yes	strong	no	3	yes
ZSM 2176/2007	14	yes	indistinct	yes	strong	yes	3	yes
ZSM 2177/2007	13	yes	indistinct	yes	strong	yes	3	yes
ZSM 2178/2007	13	yes	yes	yes	strong	indistinct	2 (+1)	yes
ZSM 2179/2007	13	yes	small	yes	strong	no	3	yes
ZSM 1506/2008	13	yes	indistinct	yes	strong	indistinct	2 (+1)	yes
ZSM 2017/2008	13	yes	yes	yes	strong	indistinct	3	yes
B. tristis								
ZSM 1704/2004 (HT)	11	yes	no	yes	indistinct	no	1	yes
ZSM 1705/2004	11	yes	no	yes	yes	indistinct	1	yes
ZSM 2146/2007	11 (indistinct)	yes	no	yes	indistinct	no	1	yes
ZSM 2147/2007	11	yes	no	yes	indistinct	no	1	yes
ZSM 2148/2007	12	yes	no	yes	no	indistinct	1	yes
ZSM 2149/2007	not countable	indistinct	no	yes	no	no	1	indistinct
ZSM 2018/2008	11	yes	no	yes	no	indistinct	1	yes
ZSM 354/2004	11	yes	no	yes	indistinct	no	1	yes
ZSM 1505/2008	12	yes	no	yes	indistinct	no	1	yes
ZSM 1706/2004	12	yes	no	yes	indistinct	no	1	yes
ZSM 1707/2004	10	yes	no	yes	no	no	1	yes
B. confidens								
ZSM 2150/2007 (HT)	13 (indistinct)	small	no	no	indistinct	yes	1	yes
ZSM 2151/2007	12 (indistinct)	small	no	no	indistinct	indistinct	2	indistinct
ZSM 2152/2007	8 (indistinct)	minute	no	no	no	yes	1 (+1)	no
ZSM 2153/2007	indistinct, not countable	yes	no	no	no	indistinct	2	indistinct
ZSM 1511/2008	indistinct, not countable	minute	no	no	no	no	2	yes

ZSM 1512/2008	12	yes	no	no	no	yes	2	yes
B. micra								
ZSM 1509/2008	11 (indistinct)	indistinct	no	yes	no	no	1	yes
ZSM 2181/2007 (HT)	11	small	no	yes	no	no	1	yes
ZSM 2183/2007	11	yes	no	yes	no	no	1	yes
ZSM 2184/2007	12	yes	no	yes	no	no	1	yes
ZSM 2185/2007	11	yes	no	yes	no	no	1	yes
ZSM 2187/2007	11	yes	no	yes	no	no	1	yes
B. sp. (aff. *micra*) (Ampombofofo) ZSM 1508/2008	not countable	indistinct	no	yes	no	no	1	indistinct

Morphological identification key to the species of the *Brookesia minima* group

The following key should allow the morphological identification of adult specimens of species in the *B. minima* group, i.e. adult *Brookesia* specimens with a SVL of 30 mm or smaller and a TL of 48 mm or smaller (SVL only overlapping with *B. lolontany,* 28 mm, which however is easily distinguished by its laterally compressed body and elongated snout). The key is preliminary given the low number of individuals known and missing hemipenial information for some species.

1. Supranasal cone present **2**
   Supranasal cone absent or ambiguous..... **11**

2. Supraocular cone present **3**
   Supraocular cone absent or indistinct **6**

3. Posterior crest on head present **4**
   Posterior crest on head absent or indistinct **5**

4. One enlarged lateral head tubercle ..... ***B. karchei***
   Three enlarged lateral head tubercles ..... ***B. desperata***

5. Pelvic spine distinct, adult male SVL > 20 mm, hemipenis ballon-like without ornamentation ..... ***B. ramanantsoai***
   Pelvic spine indistinct, adult male SVL < 20 mm, hemipenis narrow tubular with crown-like structure ..... ***B. tuberculata***

6. Dorsal spines on tail distinct ..... ***B. dentata***
   Dorsal spines on tail absent or indistinct..... **7**

7. Tail short (TAL/SVL 0.37–0.49), tail orange in life ..... ***B. micra***
   Tail longer (TAL/SVL > 0.50) **8**

8. Lateral and dorsal spines on tail always absent, inhabits western dry forests ..... ***B. exarmata***
   Dorsal spines on tail absent or insdistinct, lateral spines on tail sometimes present **9**

9. Hemipenis small, spine-like papillae on apex ..... ***B. tristis***
   Hemipenis large **10**

10. Hemipenis balloon-like without ornamentation ..... ***B. ramanantsoai***
    Hemipenis massive, bilobed, with 4 spines per lobe ..... ***B. peyrierasi***

11. Pelvic spine absent or very indistinct, body and legs very slender, hemipenis balloon-like without ornamentation ..... ***B. minima***
    Pelvic spine small but distinct, body and legs moderately robust, hemipenis very narrow with pustules on apex ..... ***B. confidens***

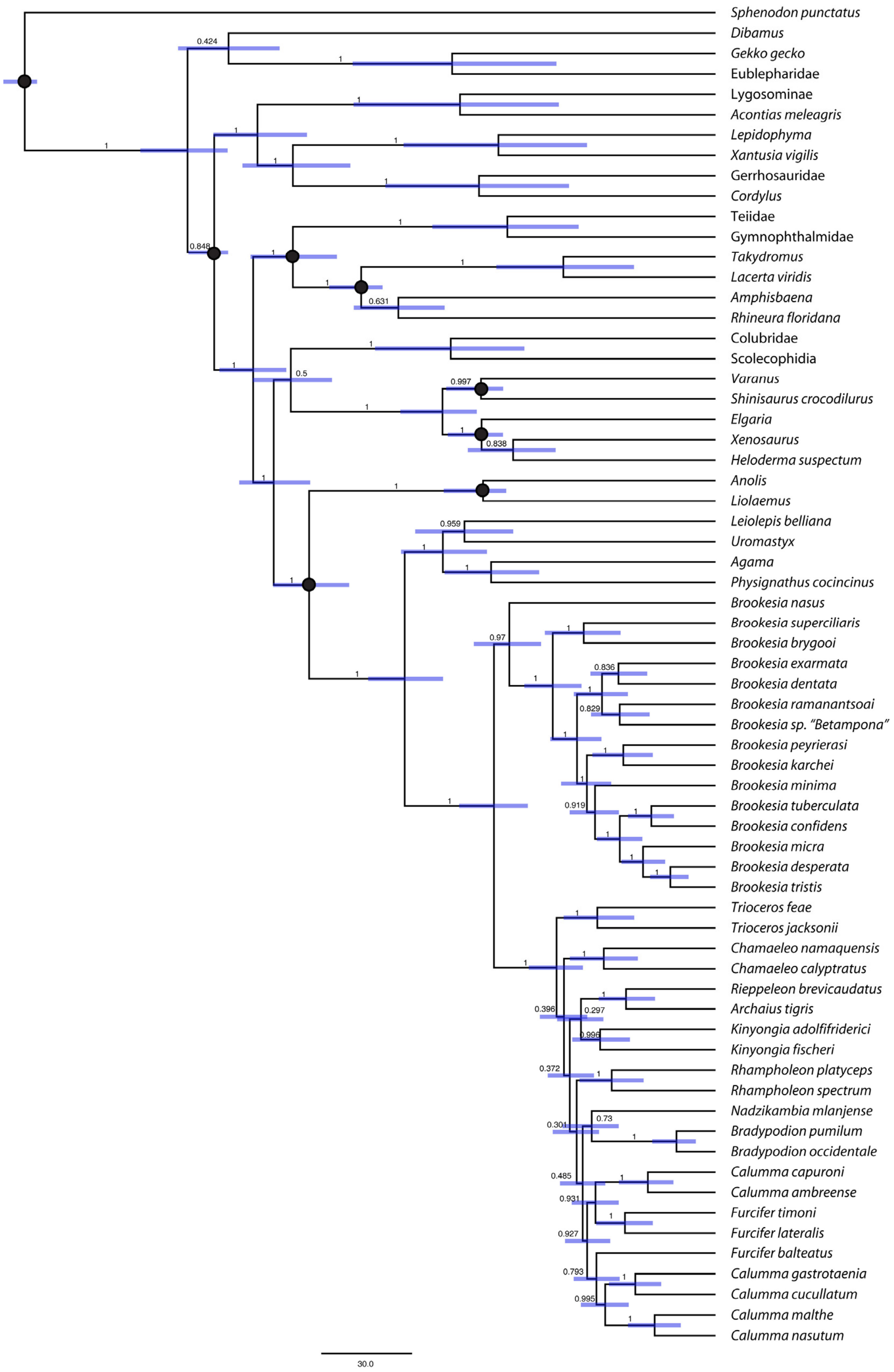

Figure S1. Full Bayesian chronogram derived from the BEAST analyses. Posterior probabilities are shown above branches, and bars represent 95% HPDs for mean date estimates. Black dots on nodes indicate calibration points (see Supplementary Methods). Units on scale are millions of years.

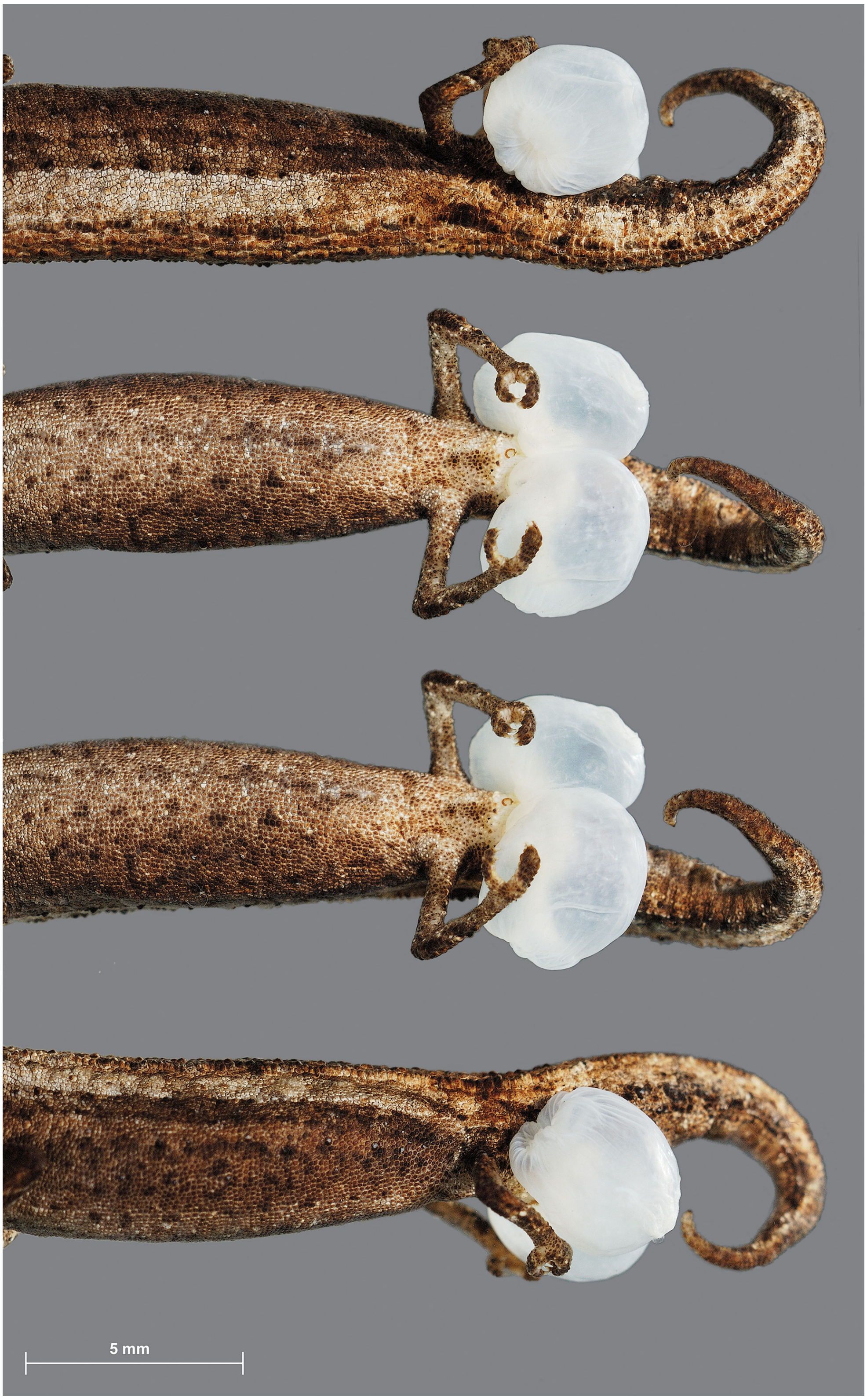

Figure S2. Male genital morphology of *Brookesia minima*.
Posterior body of preserved male specimen (lateral views and ventral view) of *B. minima* from Manongarivo (ZSM 900/2003) with everted hemipenes, showing the globular shape of hemipenes typical for *B. minima* and *B. ramanantsoai.*

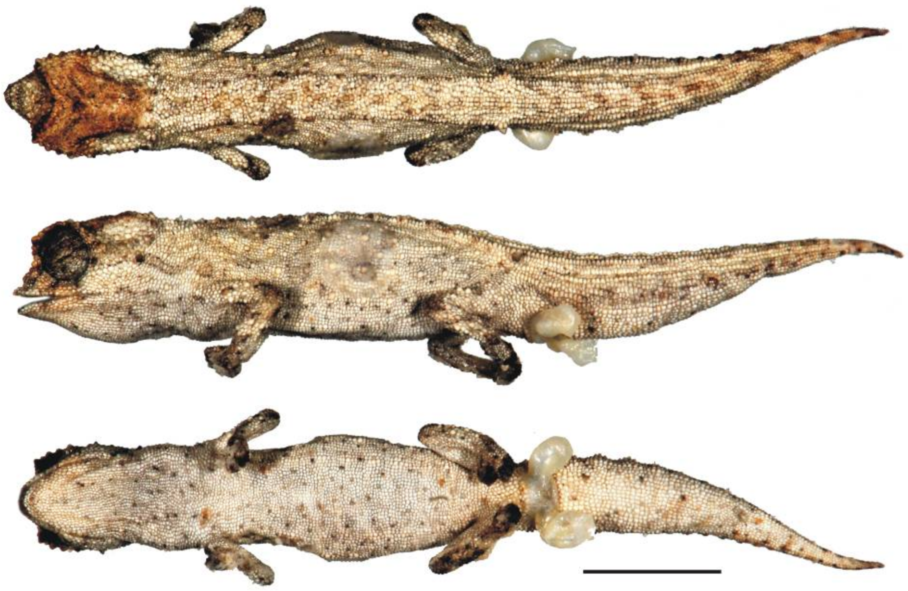

Figure S3. Dorsal, lateral and ventral views of preserved male holotype of *Brookesia tristis* (ZSM 1704/2004). Scale bar equals 5 mm.

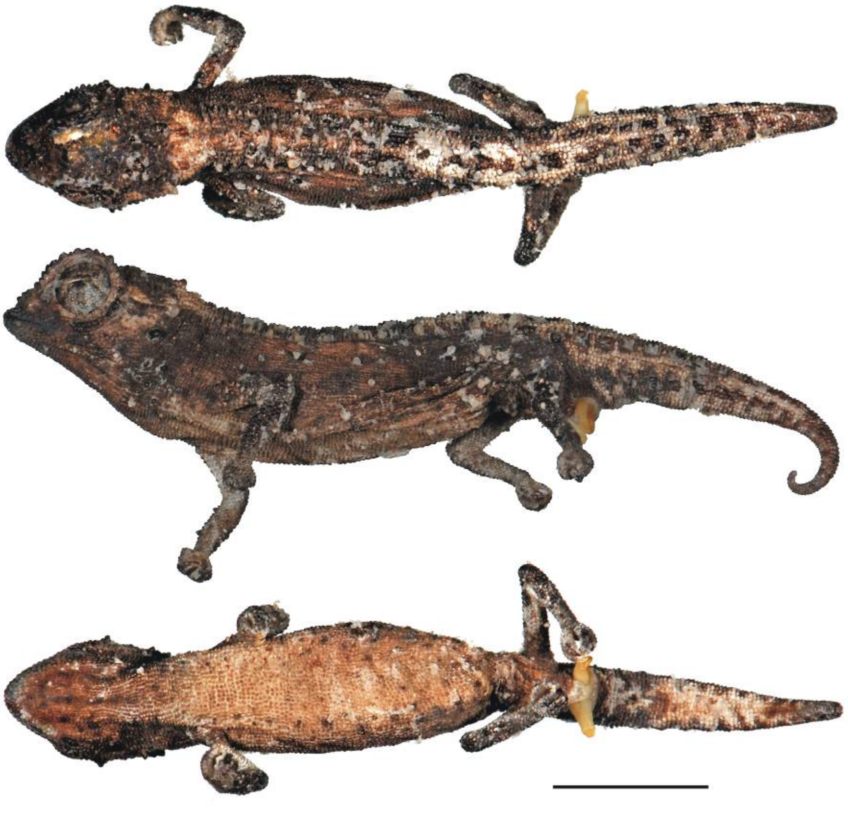

Figure S4. Dorsal, lateral and ventral views of preserved male holotype of *Brookesia confidens* (ZSM 2150/2007). Scale bar equals 5 mm.

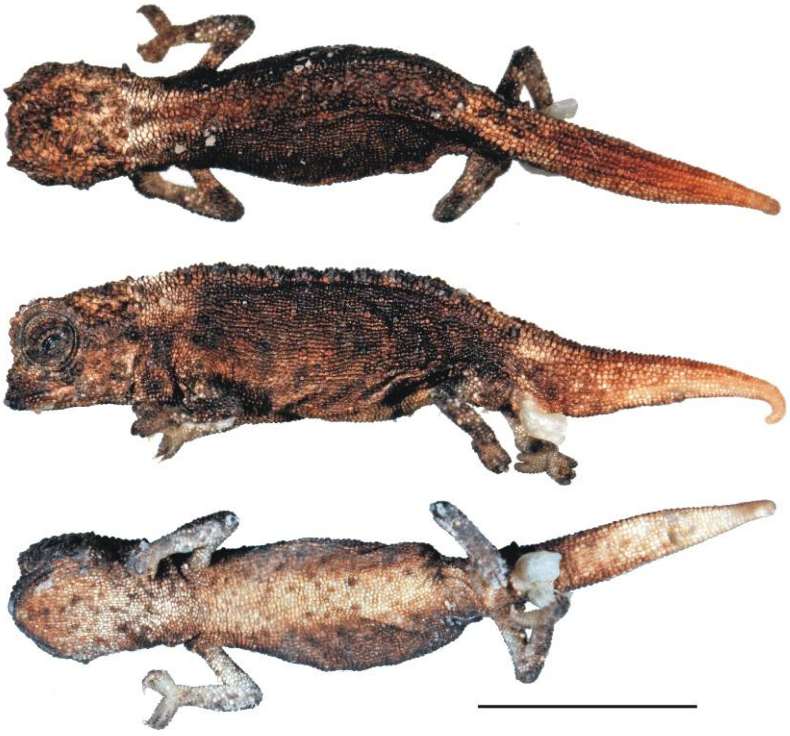

Figure S5. Dorsal, lateral and ventral views of preserved male holotype of *Brookesia micra* (ZSM 2181/2007). Scale bar equals 5 mm.

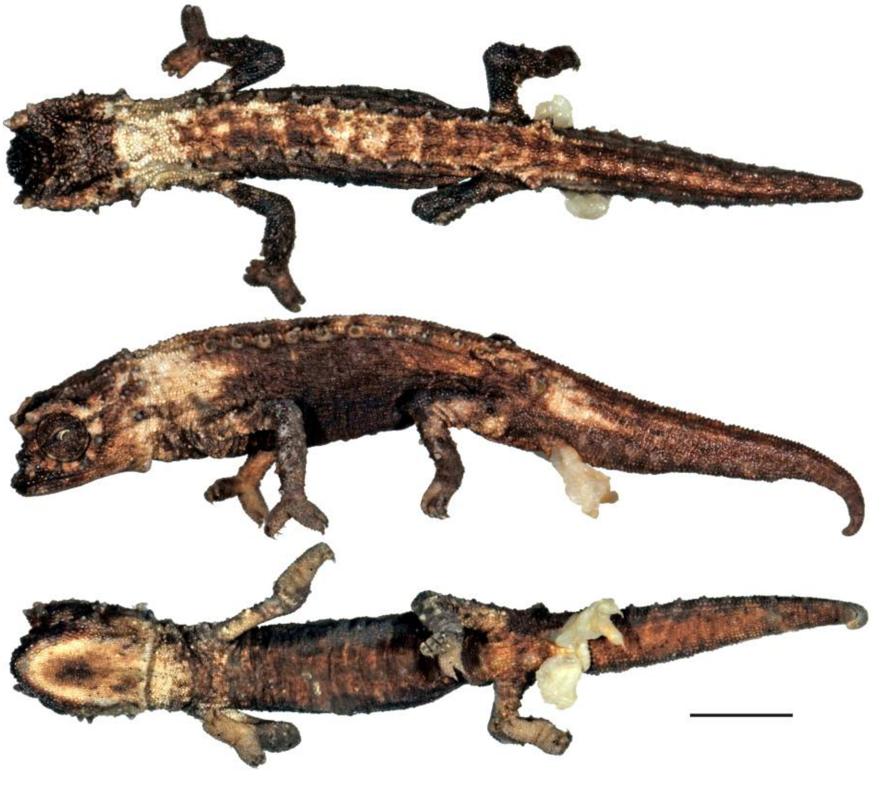

Figure S6. Dorsal, lateral and ventral views of preserved male holotype of *Brookesia desperata* from Forêt d'Ambre (ZSM 2170/2007). Scale bar equals 5 mm.
